# The Diffusion of Charged Particles in Collisional Plasmas: Free and Ambipolar Diffusion at Low and Moderate Pressures

**DOI:** 10.6028/jres.095.035

**Published:** 1990

**Authors:** A. V. Phelps

**Affiliations:** Joint Institute for Laboratory Astrophysics, National Institute of Standards and Technology and University of Colorado, Boulder, CO 80309

**Keywords:** ambipolar diffusion, boundaries, diffusion cooling, discharge maintenance, electrons, free diffusion, negative ions, positive ions, screening length, space charge

## Abstract

The interpretation of measurements of the properties of weakly ionized plasmas in terms of diffusion of electrons and ions is reviewed both critically and tutorially. A particular effort is made to tie together various aspects of charged particle diffusion phenomena in quiescent, partially ionized plasmas. The concepts of diffusion length and effective diffusion coefficient and the treatment of partially reflecting boundaries are developed in the limit of the space-charge-free motion of the electrons or ions. A simplified derivation of the screening length for space charge electric fields is followed by a review of the conventional derivation of diffusion in the ambipolar limit. A discussion of the scaling parameters of the ratio of the diffusion length to the screening length and the ratio of the diffusion length to the ion mean-free-path leads to a map used to correlate published models covering the complete range of these parameters. The models of measurements of the diffusion of electrons, several types of positive ions, and negative ions are reviewed. The role of diffusion in the decay of charged particle densities and wall currents during the afterglow of a discharge is then considered. The effects of collapse of the space charge field and of diffusion cooling are reviewed. Finally, the application of the diffusion models to a number of different discharges is discussed.

## 1. Introduction

The interpretation and planning of measurements of the properties of partially ionized, quiescent plasmas at low and moderate pressures usually requires an understanding of the loss of charged particles by diffusion to the walls. This paper is a review of models and associated experiments on the effects of space charge on the diffusion of charged particles to the walls of a discharge vessel for a wide range of discharge conditions. Previous reviews of this subject are those of Oskam [1] and of Cherrington [2]. For the most part, we will be concerned with transport perpendicular to applied electric and magnetic fields, e.g., we will not consider space charge and transport in the cathode fall or the large amplitude oscillations of electrons and ions driven by a high frequency field [3]. Phenomena which can be described by the models discussed range from nearly unperturbed or free-fall motion of electrons and ions at the low gas and charged-particle densities, through low-pressure fluorescent lamps and lasers, to the near equilibrium transport of electrons and ions in high pressure, high-temperature arcs.

This paper is both critical and tutorial in nature. It is assumed that the reader has at least a general familiarity with gas discharges and associated collision phenomena. This author still finds the review by Druyvesteyn and Penning [4] to be the most useful general discussion of gas discharges and related phenomena. Important reviews of specialized aspects include: collision phenomena, McDaniel [5]; electron energy distribution functions, Holstein [6], Allis [7] and Kumar, Skullerud, and Robson [8]; microwave discharges, Brown [3] and McDonald [9]; ion drift and diffusion in uniform electric fields, McDaniel and Mason [10]; electron transport, Huxley and Crompton [11] and Hunter and Christophorou [12]; electrical breakdown, Raether [13] and Dutton [14]; glow discharges, Francis [15]; spark channel formation, Craggs [16] and Gallimberti [17]; and gas lasers, Cherrington [2].

This discussion is divided into three major sections. In section 2 we consider the diffusion of charged particles in the absence of space charge fields. Under that topic we discuss the concept of the diffusion length, the treatment of boundary conditions, and the use of the boundary condition to calculate effective diffusion coefficients for a single type of charged particle for a wide range of gas densities. We do not discuss the very extensive work on the effects of diffusion on the electron or ion energy distributions in space-charge free and spatially uniform, applied electric fields [7,8,18–20]. The second part of the paper, section 3, is concerned with the calculation of the effects of space charge electric fields. We begin with a derivation of the Debye screening length. We then calculate the electric field strength and the ambipolar diffusion coefficient appropriate to electrical discharges with high electron densities and high gas densities. This is followed by a consideration of the range of mean-free paths, screening lengths, and diffusion lengths appropriate to various experimental conditions. The presently available theory ranges from models appropriate to the free-fall of charged particles in the absence of space charge fields to models for high gas densities and charge densities. We consider the theory of the transition from ambipolar diffusion of the charged particles at high gas densities and then at low gas densities. Next the theory is reviewed for high electron and ion densities but a range of gas densities from very low values to very large values. Finally, we discuss theoretical results which cover the whole range of gas and charge densities. In the last major section, section 4, applications of the models to specific experimental discharges are reviewed. Except in section 4.1.2 we assume that the electron energy distribution is independent of position and is determined by an applied dc or high frequency electric field or by the gas temperature.

## 2. Space-Charge-Free Diffusion

### 2.1 Diffusion Length

In this section we review models for the diffusion of charged particles in the absence of space charge fields and at high enough gas densities such that the boundary conditions are simple. Equations describing the electron and ion behavior under these conditions are [7]

$$\frac{\partial n_{q}}{\partial t}=-\nabla \cdot \Gamma_{q}+k_{i}\;n\;n_{e},$$ (1)

$$\Gamma =-D_{q}\;\nabla \;n_{q}\pm \mu_{q}\;n_{q}\;E,$$ (2)

where *q* is e for electrons or + is for positive ions and the + and − signs are for ions and electrons, respectively. Equation (1) is the continuity equation for the electron density *n*_e_ or for the density *n*_+_, where Γ*_q_* is the flux density for the electrons or ions. According to eq (1) the time derivative of the electron density is equal to the outflow of electron particle flux plus the production by electron impact ionization. This latter term contains the rate coefficient for ionization *k*_i_, the neutral atom density *n*, and the electron density *n*_e_. Equation (2) expresses the electron and ion flux densities in terms of the density gradients and the contributions from charged particle drift in any field that may be present. Here *D_q_* is the diffusion coefficient and μ*_q_* is the mobility for the electrons or ions. In this section we will assume that only a single type of charged particle is present and that electric fields are negligible.

If we combine the continuity equation and the flux equation we obtain the relation between the production term and the loss of charged particles by diffusion [7], i.e.,

$$\frac{\partial n_{q}}{\partial t}=D_{q}\left[\frac{1}{r}\frac{\partial }{\partial r}\left(r\frac{\partial n_{q}}{\partial r}\right)+\frac{\partial^{2}n_{q}}{\partial z^{2}}\right]-k_{i}\;n\;n_{q}.$$ (3)

Equation (3) is written in a form appropriate to cylindrical geometry and includes diffusion in both the radial direction and the axial direction. As shown in the next section, the electron/ion density is approximately zero at absorbing boundaries. The lowest mode solution [2,7] to eq (3) is then

$$n_{q}\left(r,z,t\right)=n_{q0}\;\exp\left(\nu \;t\right)\;\cos\left(\frac{\pi z}{L}\right)J_{0}\left(\frac{2.4r}{R}\right).$$ (4)

Substitution of eq (4) into eq (3) gives

$$\nu =k_{i}\;n-\frac{\left(nD_{q}\right)}{n\Lambda^{2}}\equiv \nu_{i}-\nu_{w},$$ (5)

where

$$\frac{1}{\Lambda^{2}}\equiv {\left(\frac{\pi }{L}\right)}^{2}+{\left(\frac{2.405}{R}\right)}^{2},$$ (6)

*ν*_i_ is the ionization frequency, and *ν*_w_ is the frequency of particle loss to the walls. Here *L* and *R* are the length and radius of the container, respectively. We will use the concept of the diffusion length A [5,7], defined for cylindrical geometry by eq (6), to characterize the size of the container throughout this report. For parallel plane geometry *R* = ∞ in eq (6) and λ*=L*/*π*, where *L* is now the electrode separation. The approximations made in assumption of zero density at the boundary are discussed in section 2.2.

### 2.2 The Effect of Boundaries

In this subsection we are concerned with a mathematical treatment of the effects of boundaries on the solution to the diffusion equation. Our approach is to solve for the transport of charged particles using the techniques of astrophysics in order to take into account the effects of charged particle reflection at the boundaries [21]. We begin with equations for the intensity *I*(θ,*z*), flux Γ(*z*), and density *n_q_*(*z*) of charged particles, taken from the theory of radiation transport [22].

$$\cos\;\theta \frac{d\;I\left(\theta ,z\right)}{dz}=-QnI\left(\theta ,z\right)+\frac{Qn}{4\pi }\int \;d\Omega^{\prime }\;I\left(\theta^{\prime },z\right)+\frac{S\left(z\right)}{4\pi },$$ (7)

Γ(z)=∫dΩI(θ,z)cosθ, (8)

and

$$n_{q}\left(z\right)=\frac{1}{\nu }\;\int \;d\theta \;I\left(\theta ,z\right).$$ (9)

Here *ν* is the speed of the particles, which are assumed to be monoenergetic. Equation (7) describes the attenuation of the beam of charged particles of intensity *I*. This intensity is assumed to be a function of the single dimension *z* and the angle θ with respect to the direction of positive *z*. The left-hand side of eq (7) gives the rate of change of the intensity with respect to position. The cos θ factor represents the projection of the intensity in the direction *z*. The first term on the right-hand side of this equation gives the loss of intensity as the result of isotropic elastic collisions with a cross section *Q*. The second term represents the effects of collisions which scatter the charged particles into the solid angle dΩ about the direction of the intensity. The 4*π* factor is the result of the assumption of isotropic scattering. The last term in this equation represents production of charged particles, which is also assumed to be isotropic. Equations (8) and (9) are expressions for the current density in the *z* direction and for the density of charged particles in terms of integrals over the intensity.

Equations (7)–(9) are solved using what is known as the two-stream approximation. In the formulation from Chandrasekhar [22], the two streams are assumed to lie on the surface of a cone making an angle θ*_i_* with the positive *z* direction. With this approximation one obtains equations for *I*_+_, representing the intensity in the +*z* direction, and *I*_−_, representing the intensity in the −*z* direction which are

$$\cos\;\theta_{i}\frac{dI_{+}}{dz}=-\frac{Q\;n\;I_{+}}{2}+\frac{Q\;n\;I_{-}}{2}+\frac{S\left(z\right)}{4\pi },$$ (10)

and

$$-\cos\;\theta_{i}\frac{dI_{-}}{dz}=-\frac{Q\;n\;I_{-}}{2}+\frac{Q\;n\;I_{+}}{2}+\frac{S\left(z\right)}{4\pi }.$$ (11)

In this section only the subscripts + and − refer to direction relative to the *z* axis and not to the sign of charge. The flux or particle current density and the particle density are then given by

$$\Gamma \left(z\right)=2\pi \;\cos\;\theta_{i}\left(I_{+}-I_{-}\right),$$ (12)

and

$$n_{q}\left(z\right)=\frac{2\pi }{\nu }\left(I_{+}+I_{-}\right).$$ (13)

On the right-hand side of eq (10) we first have the loss of the *I_+_* component of intensity due to collisions. The second term is the gain due to collisions of the other component of the intensity. Finally one has the source term. Equation (11) is a similar equation giving the continuation of the negatively-directed component. Equations (12) and (13) give expressions for the current density and the particle density in terms of the positive and negative components of the intensity. It should be noted that with the choice of cos θ*_i_* = 1/*√*3, the equations for the flux and density in the two stream approximation to the angular distribution are identical to those obtained with the first two terms of the spherical harmonic expansion [21].

Our next step [21] is to rewrite eqs (10)–(13) in forms similar to those of eqs (1) and (2). Adding eqs (10) and (11) for *I_+_* and *I_−_* gives

$$\cos\theta_{i}\frac{d\left(I_{+}-I_{-}\right)}{dz}=\frac{S\left(z\right)}{2\pi },$$ (14)

while subtracting the equations yields

$$\cos\theta_{i}\frac{d\left(I_{+}+I_{-}\right)}{dz}=-Qn\left(I_{+}-I_{-}\right).$$ (15)

Term by term comparison of eqs (14) and (15) with eqs (1) and (2) in the limits of *E*=0 and d*n_q_*/d*t* = 0 leads one to define a diffusion coefficient for this approximation by

$$D_{q}=\cos^{2}\theta_{i}\frac{\nu }{Qn}=\frac{\nu }{3Qn}=\frac{\lambda_{q}\nu }{3},$$ (16)

where λ*_q_* is the mean-free-path for the particle of charge type *q*. If one adopts a Gaussian weight factor of cos θ*_i_* = 1/√3 as discussed by Chandrasekar [22], then one obtains the familiar form for the diffusion coefficient [21] given in the second and third equalities of eq (16).

We now come to the part of the derivation directly concerned with boundary conditions and follow the treatment of Chantry, Phelps, and Schulz [21]. We express the boundary condition at *z* = 0 by writing the intensity leaving the boundary *I_+_* as equal to a reflection coefficient times the intensity *I*_−_ arriving at the boundary, i.e., *I*_+_ = *ρI*_−_. Substitution of this relationship into eqs (13) and (15) yields the magnitude of the normalized slope of the density of charged particles at the boundaries, i.e.,

$$\left|\frac{1}{n_{q}}\frac{dn_{q}}{dz}\right|=\frac{Qn}{\cos\theta_{i}}\frac{\left(1-\rho \right)}{\left(1+\rho \right)}=\frac{\surd 3\left(1-\rho \right)}{\lambda_{q}\left(1+\rho \right)}\equiv \frac{1}{\ell_{q}}.$$ (17)

Equation (17) is often called the Milne boundary condition [23].

**Figure 1:**
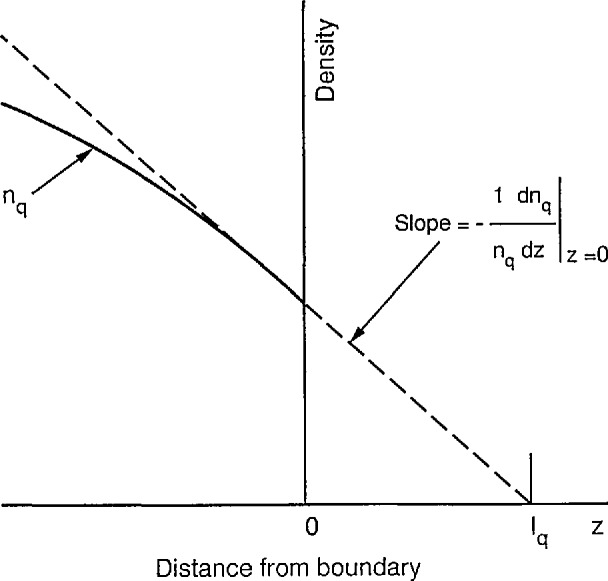
Boundary condition for electron and ion densities.

The meaning of the various terms in the boundary condition represented by eq (17) are illustrated in figure 1. The dashed line shows the value of (1/*n*)(d*n*/d*z*) at *z* = 0. The intercept of this line with the abscissa occurs at a distance beyond the boundary equal to *ℓ_q_* = (1 + *ρ*)λ*_q_*/√3(1−*ρ*). In the limit of zero reflection *ℓ_q_* =.λ*_q_*/√3 A more detailed calculation [23,24] leads to the conclusion that for planar geometry the √3 in eq (17) should sometimes be replaced by a number between 1.41 and 1.5 depending on the reflection coefficient.

### 2.3 Effective Diffusion Coefficient

We now consider the boundary condition appropriate to the problem of diffusion in an infinite cylinder and in the limit of a steady-state solution. We follow the treatment of McCoubrey [25]. The fundamental mode solution to eq (3) is the well-known zero-order Bessel function, i.e., *n*(*r*)*=J*_0_(*jr/R*). Substitution of this solution into the boundary condition, eq (17), yields the relation between the quantity *j*, the radius of the cylinder *R*, the mean-free path λ*_q_*, and the reflection coefficient *ρ*

$$\frac{jJ_{1}\left(j\right)}{R\;J_{0}\left(j\right)}=\frac{\surd 3\left(1-\rho \right)}{\lambda_{q}\left(1+\rho \right)}.$$ (18)

Note that *j* is smaller than the value of 2.4 which is the first root of *J*_0_(*j*). For parallel plane geometry the left hand side of eq (18) becomes *j* tan (*j*)/*L*. Substitution of eq (18) into eq (3) yields the condition for a steady-state discharge, i.e., the discharge “maintenance” condition,

$$k_{i}\;n=\frac{D_{q}j^{2}}{R^{2}}\equiv \frac{D_{sq}\left(\Lambda /\lambda_{q},\rho \right)}{\Lambda^{2}},$$ (19)

or for cylindrical geometry

$$n\;D_{sq}\left(\Lambda /\lambda_{q},\rho \right)={\left(\frac{j\left(\Lambda /\lambda_{q},\rho \right)}{2.405}\right)}^{2}n\;D_{q}.$$ (20)

In the second equality of eq (19), we have chosen to express the results in terms of an effective diffusion coefficient *D*_s_*_q_*.

**Figure 2:**
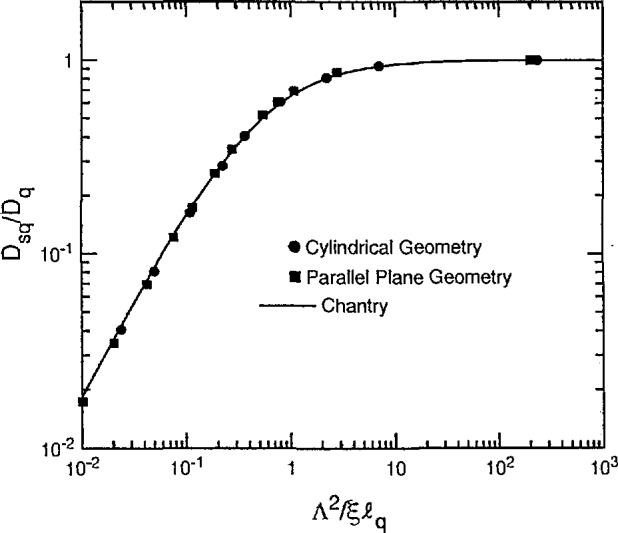
Normalized diffusion coefficient for a single type of particle.

Chantry [24] has recently proposed a simple empirical approximation to eq (20) which is

$$\frac{D_{sq}}{D_{q}}=\frac{1}{\left(1+\frac{\xi \;\ell_{q}}{\Lambda^{2}}\right)}$$ (21)

where ξ is the volume of the discharge chamber divided by its surface area and the “extrapolation length” *ℓ _q_* is defined by eq (17). This relation is useful for arbitrary geometry, e.g., ξ/Λ is 2.405/2 for infinite cylindrical geometry and *π*/2 for parallel plane geometry. The curve and points of figure 2 show plots of the ratio *D*_s_*_q_*/*D_q_* as a function of the ratio Λ^2^/ξ*ℓ_q_* for *ρ* = 0. The solid curve is eq (21) while the circles are calculated from eq (20) for cylindrical geometry [25]. The squares are calculated from the corresponding equation for parallel-plane geometry. The agreement among the values shown by the points and the curve is evidence of the success of Chantry’s approximation. Figure 2 shows that the effective diffusion coefficient *D*_s_*_q_* used to describe the loss of charge particles in eq (19) approaches *D_q_* as the container size increases and the extrapolated length *ℓ_q_* and mean-free-path *X_q_* decrease. At low values of Λ^2^/ξ*ℓ_q_* the value of *D*_s_*_q_* decreases below the high pressure limit.

**Figure 3:**
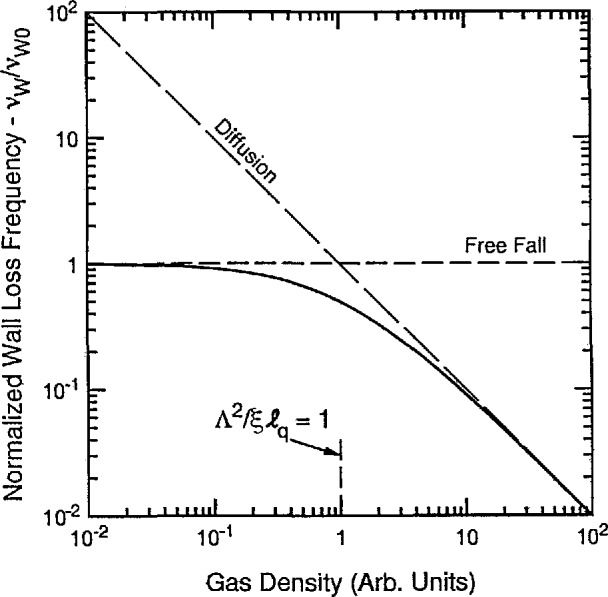
Apparent diffusion coefficient vs gas density.

Since a cursory glance at figure 2 makes it appear that charged particle losses to the wall become much less important as the mean-free path becomes longer, we show in figure 3 a plot of the wall loss frequency *ν*_W_, defined in eq (5) and normalized to its value at low densities *ν*_W0_ as a function of the gas density. The horizontal scale for figure 3 is actually Λ^2^/ξ*ℓ_q_*, which is proportional to gas density through 1/*ℓ_q_*. At low gas densities the loss frequency *ν*_W_ = *ν*_W0_ is independent of density, i.e., collisions are negligible and the charged particles move freely to the wall. At large gas densities the wall loss frequency varies inversely with gas density and is given by the *v*_W_ = *D_q_*/Λ^2^ = λ*_q_v/*3Λ^2^, where D*_q_* and λ*_q_*, vary inversely with density. Figure 3 shows that the transition between the two regions occurs when Λ^2^/ξ*ℓ_q_* is equal to 1. Note that the transition seen here between the two limiting forms of loss to the boundary is often observed for neutral free radicals in chemical reactors [26].

## 3. Effects of Space Charge on Diffusion of Electrons and Ions

We begin this section with a simple formulation of the length which characterizes space charge phenomena in steady-state plasmas, i.e., the screening length. We then consider limiting forms of the theory of effects of the self-consistent electric field or space charge field on the steady-state diffusion problem. Finally, we review the general solutions and compare empirical approximations to the results of numerical models.

### 3.1 Screening Length

The screening or shielding length [27] characterizes the distance over which an electric potential change influences the charge distribution. The spatial variation of the potential *V*(*r*,*z*) is related to the positive ion and electron densities by Poisson’s equation, i.e.,

$$\nabla^{2}\;V\left(r,z\right)=-\frac{e}{\epsilon_{0}}\left(n_{+}-n_{-}-n_{e}\right).$$ (22)

Note that here and in the rest of this paper, the subscripts + and − refer to positive and negative ions. Plasmas with several types of ions, including negative ions, will be considered in section 3.4. For the purposes of this discussion, we assume one dimensional geometry and that either positive ions or electrons are dominant and of nearly uniform density. The solution to eq (22) is then

$$\Delta V=\frac{n_{q}e}{2\epsilon_{0}}\Delta z^{2},$$ (23)

where *n_q_* is the density of charged particles. We then characterize this change by the distance required for the potential energy to change by an energy equal to half that of the particle temperature, i.e., by *eΔV=kT_q_*/2. This distance is called the screening or Debye length λ_D_*_q_* and is given by

$$\lambda_{D_{q}}^{2}=\frac{\epsilon_{0}kT_{q}}{e^{2}n_{q}}=\frac{kT_{q}}{8\pi \;Ry\;a_{0}\;n_{q}},$$ (24)

where in the second equality λ_d_*_q_* is in m, *kT_q_* is the charged particle temperature in eV, *n_q_* is in m^−3^, *Ry* = 13.6 eV, and *a*_0_=5.29 × 10^−10^ m. Note that the screening length for electrons and ions differ because of differing temperature and densities. We will be primarily concerned with the electron screening length λ_De_.

The screening length relation is used for scaling the results of space charge controlled motion and can also be derived from the detailed theories, such as that of Allis and Rose [28], to be discussed in section 3.3.2.

### 3.2 Ambipolar Diffusion Theory for High Electron, Ion, and Gas Densities: (Λ/λ_De_>>1 and Λ/λ_+_>>1)

We now consider the effects of space charge in the limits for which the screening length and the mean-free path for positive ions λ_+_ are much smaller than the diffusion length Λ, i.e., in the region of high electron, ion, and gas densities but negligible electron-ion recombination, etc.. This problem was solved many years ago by Schottky [29] and the resultant diffusion is termed ambipolar diffusion. One begins with expressions for the flux of electrons and ions:

$$\Gamma_{e}\left(z\right)=-D_{e}\;\nabla \;n_{e}-\mu_{e}\;n_{e}\;E_{s},$$ (25)

and

$$\Gamma_{+}\left(z\right)=-D_{+}\;\nabla \;n_{+}+\mu_{+}\;n_{+}\;E_{s}.$$ (26)

Equation (25) shows that in the limits considered here the electron flux is equal to the negative of a diffusion term minus a term representing the drift of electrons in response to the electric field *E*_s_ generated by the difference in the electron and ion densities, i.e., the space-charge field. A similar expression is given in eq (26) for the positive ions. Note that the sign of this mobility or drift term has been changed as is appropriate to the change in sign of the charge. For the steady-state problems of interest to us the electron flux is equal to the positive ion flux so that there is no build-up of charge difference, i.e., Γ_e_ is equal to Γ_+_. Next it is assumed that *n*_e_*−n_+_<<n*_e_ or *n_+_*, i.e., the quasi-neutrality assumption. With these assumptions eqs (25) and (26) yield an expression for the space charge electric field:

$$E_{s}=-\frac{\left(D_{e}-D_{+}\right)}{\left(\mu_{e}+\mu_{+}\right)}\frac{\nabla \;n_{e,i}}{n_{e,i}}\approx -\frac{D_{e}}{\mu_{e}}\frac{\nabla n}{n}.$$ (27)

In the second equality we assumed that *D*_e_>*>D_+_* and μ_+_ >>μ_+_, as is usually the case. In this limit the electric field is determined entirely by the electrons.

**Figure 4:**
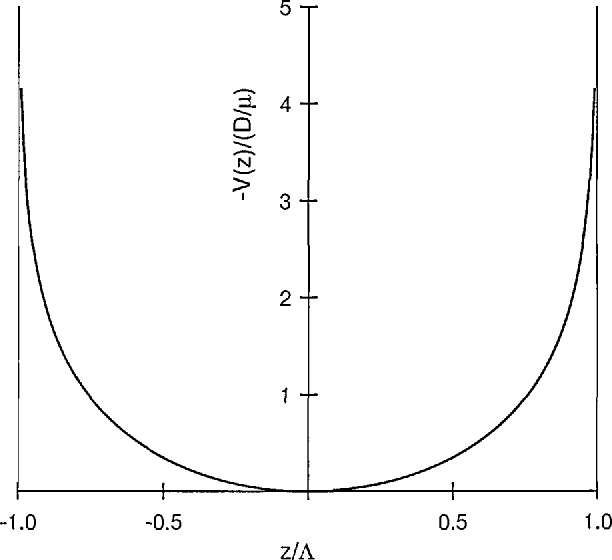
Ambipolar space charge potential as seen by electrons.

Substitution of eq (27) for the electric field into eq (25) shows that the two terms on the right-hand side essentially cancel each other. Physically this means that the electron diffusion current is balanced rather closely by the electron drift current and that the electrons essentially sit in a potential well. The potential well appropriate to parallel plane geometry is drawn in figure 4. It is based on the approximate solution for an absorbing boundary, i.e., that *n*(*z*) *= n*_0_ cos(*πz*/*l*) so that *V*(*z*)=(*D*_e_/µ_e_) In [cos(*πz/L*)]. Note that this potential becomes infinite at the boundary so that the electrons cannot escape. The resolution of this difficulty through departures from the ambipolar limit, i.e., the presence of a space charge sheath, near the boundary is treated in the models of section 3.3.

The final expression for the positive ion flux is

$$\Gamma_{+}=-\frac{\left(\mu_{e}D_{+}+\mu_{+}D_{e}\right)}{\left(\mu_{e}+\mu_{+}\right)}\nabla \;n_{+}=-D_{a}\;\nabla \;n_{+},$$ (28)

where for μ_e_>>μ_+_

$$D_{a}\approx \left(1+\frac{D_{e}/\mu_{e}}{D_{+}/\mu_{+}}\right)D_{+}\approx \left(1+\frac{T_{e}}{T_{+}}\right)D_{+}.$$ (29)

The effective diffusion coefficient *D*_a_ is known as the ambipolar diffusion coefficient. Equation (29) shows that when the electron *D*_e_/μ_e_ ratio is much greater than the positive ion *D_+_/*μ*_+_* ratio, as in an active discharge, then the ambipolar diffusion coefficient is much greater than the positive ion diffusion coefficient. The motion of the positive ions in the potential shown in figure 4 is that of a continually accelerating drift. We will see later how this model fails as one approaches the boundaries of the discharge tube.

The electron and ion mobilities and diffusion coefficients have been assumed constant in eqs (25)–(29). The effects of an *E/n* dependent ion mobility have been considered by Frost [30] and by Zakharova, Kagan, and Perei’ [31]. Other geometries, such as coaxial cylinders, have been considered [32–34]. The second equalty in eq (29) assumes that the electron and ion energy distributions are Maxwellian at temperatures of *T*_e_ and *T*_i_, respectively. When the electron energy distribution is not Maxwellian the concept of a temperature is only approximately correct and it is better to retain *eD*_e_*/*μ_e_ from the first equality of eq (29) rather than use *kT*_e_ [35]. The effects of a non-Maxwellian ion energy distribution apparently have not been discussed in this context.

Two useful limits of eq (29) are the thermal equilibrium limit, i.e., *T*_e_ *= T_+_*, and the active discharge limit, i.e., *T*_e_>>*T*_+_. The thermal equilibrium limit is often applicable in the afterglow of pulsed discharges [1,5] and results in

$$D_{a}=2\;D_{+}.$$ (30)

This subject will be considered further in section 3.5.1. The active discharge limit often applies in the positive column, etc., [4] and results in

$$D_{a}=\frac{D_{e}}{\mu_{e}}\mu_{+}\approx \frac{T_{e}}{T_{+}}D_{+}.$$ (31)

This limit will be considered in more detail in section 4.1.1.

### 3.3 Transitional Ambipolar Diffusion

#### 3.3.1 Map of Λ/λ_De_ and Λ/λ_+_ Space

**Figure 5:**
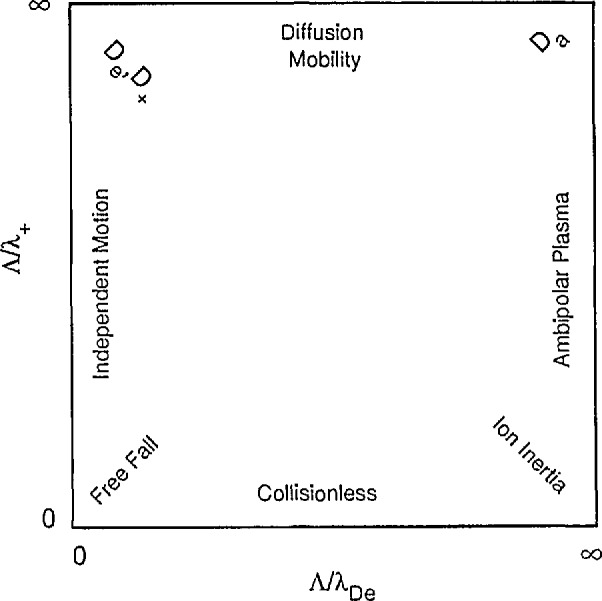
Map of transitional ambipolar diffusion parameters and solutions.

In order to correlate various experimental and theoretical investigations of ambipolar diffusion, we have shown in figure 5 a schematic of the range of diffusion lengths, positive ion mean-free-paths, and screening lengths considered in the theories we will discuss, i.e., 0 < Λ/λ_De_< ∞ and 0 < Λ/λ_+_< ∞. The space-charge free limit resulting in independent particle transport which we considered in section 2 corresponds to moving downward along the left-hand side of this square. At the upper left corner we have the independent diffusion of the electrons and the positive ions. At the lower left corner we have the collision-free and field-free, i.e., “free fall,” motion of the ions and electrons to the boundary. The ambipolar diffusion limit considered in section 3.2 occurs in the upper right-hand corner of the square, where both the mean-free-path and the screening length are short compared to the diffusion length. The transition from free diffusion to ambipolar diffusion at high gas densities which occurs along the top side of the square is discussed in section 3.3.2. This is followed in section 3.3.3 by a summary of theory of the collisionless transition along the bottom of the square from space charge free to space charge dominated or ambipolar motion. This theory includes inertial effects which describe the acceleration of ions in the absence of collisions. The theory including both collision and inertia terms is summarized in section 3.3.4 for the limit of λ_De_<< Λ, i.e., along the right-hand side of the square. Section 3.3.5 summarizes theoretical results applicable to all of figure 5. Positive ion diffusion for Λ/λ_+_>> 1 is discussed in section 3.3.6.

#### 3.3.2 The Transition from Free to Ambipolar Diffusion at High Gas Densities: Λ/λ_+_≫1 and 0<Λ/λ_De_< ∞

We now consider the transitional ambipolar diffusion problem for electrons and positive ions corresponding to experiments in which the mean-free-path is much smaller than the diffusion length, and in which the electron screening length may vary from much smaller than the diffusion length to much larger than the diffusion length. This problem was first solved by Allis and Rose [28] and we will summarize their results. The applicable equations are

$$\Gamma_{e}\left(z\right)=-D_{e}\;\nabla \;n_{e}-\mu_{e}\;n_{e}\;E_{s},$$ (25)

$$\Gamma_{+}\left(z\right)=-D_{+}\;\nabla \;n_{+}+\mu_{+}\;n_{+}\;E_{s},$$ (26)

$$\nabla \cdot \Gamma_{e,+}=k_{i}\;n\;n_{e},$$ (32)

and, since *n*_−_= 0, eq (22) becomes

$$\nabla^{2}\cdot V\left(r,z\right)=-\nabla \cdot E_{s}\left(r,z\right)=-\frac{e}{\epsilon_{0}}\left(n_{+}-n_{e}\right),$$ (33)

with the boundary conditions for absorbing walls of

$$n_{e}\left(R\right)\approx n_{+}\left(R\right)\approx 0.$$

Equations (25) and (26) repeat the continuity equations for electrons and ions. Equation (32) is the steady-state form of eq (1) for positive ions (*q = +*), while eq (33) is eq (22) with *n*_−_ = 0. The mobility μ and diffusion *D* coefficients are usually treated as independent of position. The boundary conditions require that the electron and positive ion densities are zero at the generalized boundary at *R*. Following Allis and Rose [28] we expressed the results in terms of an effective diffusion coefficient *D*_se_ for electrons defined for a steady-state discharge such that

$$k_{i}n\;n_{e0}=\nu_{w}\;n_{e0}\equiv \frac{n\;D_{\mathrm{se}}\;n_{e0}}{\Lambda^{2}},$$ (34)

where *n*_eD_ is the electron density at the center of the discharge. The ratio *D*_se_/*D*_e_ is a measure of the change in the diffusion loss of electrons caused by the ambipolar electric fields. Note that some authors, e.g., Ingold [36] and Cohen and Kruskal [37], express the results of the theory in terms of the ionization frequency *ν*_i_ *≡ k*_i_ *n* required to maintain the discharge, or in terms of its equal for a steady-state discharge, the frequency of electron loss to the walls *ν*_W_. Two important limits of the solutions to eqs (25), (26), (32), and (33) have been discussed in sections 3.2 and 2 of this report. When λ*_D_*_e_<< Λ the ambipolar limit applies and *d*_se_ = *D*_a_. When λ*_D_*_e_>>Λ, free diffusion of the electrons occurs and *D*_se_ = *D*_e_. According to the expression for the ambipolar diffusion coefficient given in eq (29), the ratio of the ambipolar diffusion coefficient to the free electron diffusion coefficient *D*_se_/*D*_e_ for an active discharge (*T*_e_*>>T_+_*) is equal to the ratio of the positive-ion mobility to the electron mobility. Typical experimental values *D*_se_/*D*_e_ are from 10^−4^ to a few times 10^−2^.

A number of solutions have been obtained for the transition from free to ambipolar diffusion at high gas densities. One of the earliest was that reported by Holstein [38], who replaced the radius of the tube for electron loss by the radius minus a plasma sheath thickness very nearly equal to the screening length. A second approximation uses the idea that since electron and ion densities have the same spatial distribution in the limits of large and small screening lengths one can assume that the ratio of the electron and ion densities is everywhere constant [28,39,40]. Since these solutions are not accurate at intermediate values of λ*_D_*_e_/Λ, one must beware of this popular approach. Numerical solutions of eqs (25), (26), (32), and (33) were obtained by Allis and Rose [28] for parallel plane geometry. More recently, numerical techniques have been used for 0 < Λ/λ*_D_*_e_ < ∞ in the Λ/λ_+_⪢ limit by Kregel [41] as part of time dependent solutions and by Ingold [36] as part of steady-state solutions for 0 < Λ/λ_+_ < ∞. Models based on the separation of the plasma into a central region matched to a sheath have been extended and refined by several authors [28,37,42,43]. The more recent results agree well with the numerical results of Allis and Rose [28]. Another approach uses power series approximations to the electron and positive ion densities in parallel-plane geometry [44,45] and cylindrical geometry [46]. For reasons which are not understood, the latter results [46] for D_se_ are lower than the numerical results [28]. An analytic solution for an isothermal plasma, i.e., *T_+_ = T*_e_, by Numano [47] agrees with Allis and Rose [28] for a limited range of μ_+_/μ_e_.

**Figure 6:**
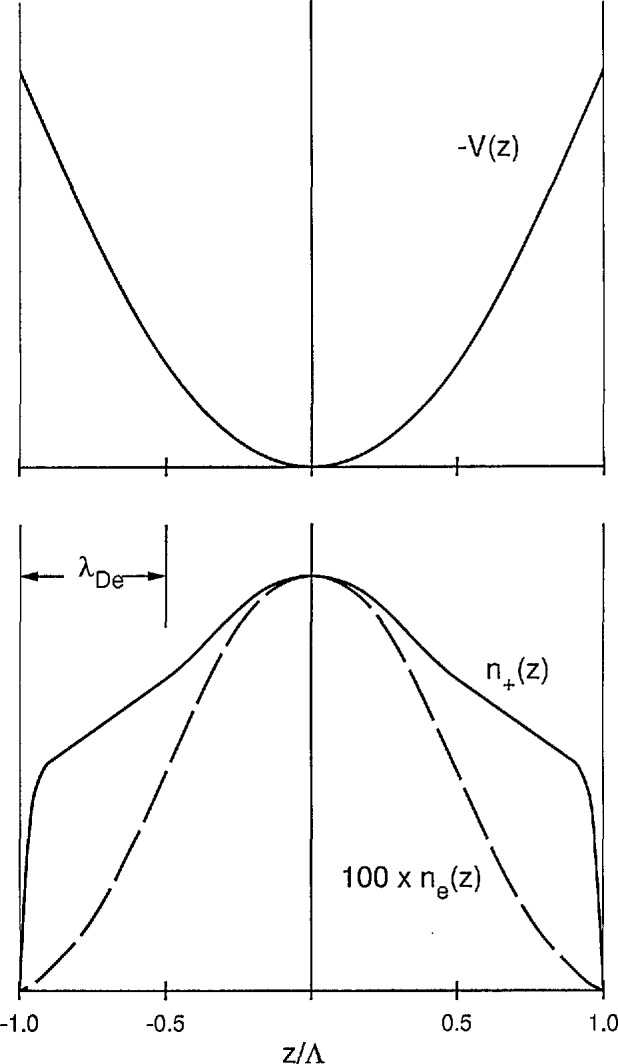
Schematic spatial distributions of potential and particle densities.

Figure 6 shows schematics of spatial distributions for the electric potential and the electron and positive-ion densities obtained by the numerical techniques [28]. The upper curve shows a schematic of the space-charge potential *V*(*z*) as a function of distance for parallel-plane geometry. In the central portion of the gap near *z* = 0 the potential is the same as shown in figure 4, i.e., it is approximately parabolic near its origin and increases with distance from the origin at a rate determined by the *D/*μ value for the electrons. At a distance from the electrodes of the order of the screening length for the electrons, the potential variation changes character and increases less rapidly, reaching a finite value at the electrodes. This finite potential allows the electrons to escape over the potential barrier and reach the wall. The transition region near the wall is known as the ion sheath, because of the dominance of positive ions. In the lower part of figure 6 we show a schematic of the calculated positive ion and electron densities. Note that the electron density curve has been multiplied by 100. The electron density decreases more rapidly than given by the cosine function characteristic of free diffusion or of ambipolar diffusion, and it approaches zero at a distance from the wall of the order of the electron screening length. The positive ion density on the other hand is much flatter and decreases rapidly only at distances from the wall such that ion diffusion becomes more important than the ion drift in the space charge electric field which dominates over most of the volume.

**Figure 7:**
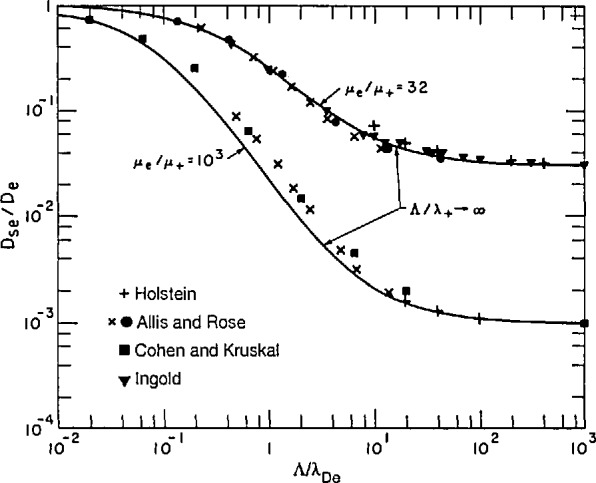
Normalized diffusion coefficients vs Λ/λ_De_ for λ*_+_/*Λ<<1.

In figure 7 we have compared some of the theoretical results for the effective diffusion coefficient as a function of the ratio of the diffusion length to the electron screening length. The simple approximation due to Holstein is indicated by +’s. We see that it is close to the other points when the ratio of the electron mobility to the ion mobility is relatively small, as in the case of *μ*_−_/*μ*_+_ = 32. Holstein’s approximation is much worse for larger ratios of mobility. The numerical results of Allis and Rose are indicated by the X ‘s and solid circles and show the smooth transition from values *D*_se_*/D*_e_ near unity at low values of the ratio Λ/λ_De_ to values equal to the ratio of mobilities of positive ions and electrons at high ratios Λ/λ_De_. Similar ratios have been calculated numerically by Cohen and Kruskal [37], solid squares, and by Ingold [36], inverted triangles. The solid curves are empirical fits to these calculations and will be discussed later.

#### 3.3.3 Effects of Space Charge at Low Gas Densities: λ_+_/Λ>>1 and 0<Λ/*D*_e_< ∞

We now turn to the transition between free “diffusion” and am-bipolar “diffusion” which occurs at low gas densities, that is when λ_+_/Λ >> 1 and the charged particle motion approaches free-fall in the space charge electric field. The long mean free paths mean that the collisional-equilibrium model of charged article motion eq (2) will have to be replaced by a model in which the ion velocity distribution is no longer a function of just the local electric field. The screening lengths vary from very small values to large values compared to the diffusion length. In the Λ_+_/Λ>> 1 limit, the frequency of charged particle loss or reciprocal lifetime is independent of gas density as for low Λ/λ_+_ in figure 3. Nevertheless, for the sake of unity we will express the results in terms of an effective diffusion coefficient for electrons relative to the free diffusion coefficient. Here we are concerned with solutions obtained along the lower side of the square of figure 5.

We follow the treatments of Tonks and Langmuir [48] for λ_De_/Λ<< 1 and the treatment of Self [49] for arbitrary values of Λ/λ_De_. Another discussion of related models is given by Dote and Shimada [50]. For simplicity we discuss only the one dimensional solution. The potential form of Poisson’s equation, eq (33), is the basic equation for this treatment. The electrons are assumed to be trapped in the electrostatic potential and to obey the Boltzmann equation for their distribution in that potential, i.e., *n*_e_ = *n*_0_ exp[*−eV*(*z*)/*kT*]. The density of positive ions is

$$n_{+}\left(z\right)=\int_{0}^{z}\frac{dy\;k_{i}\;n\;n_{e}\left(y\right)}{\nu_{+}\left(y,z\right)}.$$ (35)

Here ν_+_(*y*,*z*) is the velocity a positive ion acquires in moving of its point of production at *y* to the point *z*. The equations for the electron and ion fluxes given by eqs (25) and (26) are no longer adequate and are replaced by

$$\Gamma_{e}=-D_{e}\;\nabla \;n_{e}-\mu_{e}\;n_{e}\;E_{s}-\frac{n_{e}}{m_{e}\nu_{\mathrm{en}}}\nabla \left(m_{e}\nu_{e}^{2}/2\right),$$ (36)

and

$$\Gamma_{+}=-D_{+}\;\nabla \;n_{+}+\mu_{+}\;+n_{+}\;E_{s}-\frac{n_{+}}{m_{+}\nu_{+n}}\nabla \left(m_{+}\nu_{+}^{2}/2\right).$$ (37)

Here *ν*_en_ and *ν*_+n_ are the collision frequencies for electrons and ions with the neutral gas and are defined for practical purposes by their relation to the corresponding mobilities, i.e., *ν_j_* ≡ *e*/(*m_j_* μ*_j_*). Equations (35)–(37) were obtained from the first two velocity moments of the Boltzmann equations for electrons and ions. The last terms on the right-hand sides of eqs (36) and (37) are called the inertia terms and become important when λ_e_ or λ_+_ becomes comparable with the dimensions of the plasma region under consideration [36, 51–55].

For the conditions of interest here the electrons are sufficiently close to equilibrium in the potential well that the inertia and flux terms of eq (36) can be neglected. On the other hand, at the low gas densities considered in this section an approximate solution to eq (37) is obtained by equating the last two terms on the right-hand side to obtain

$$\frac{m_{+}\nu_{+}^{2}}{2e}=\int_{z_{1}}^{z_{2}}\;dz\;E_{s}\left(z\right)=V\left(z_{1}\right)-V\left(z_{2}\right).$$ (38)

Here the electron starts at rest at *z*_1_. Equation (38) is simply the conservation of energy in the collisionless limit. When eq (38) is solved for *ν*_+_ and used in the integral form of eq (35) for *n_+_*, the one dimensional form of eq (33) becomes

$$\frac{d^{2}V\left(z\right)}{dz^{2}}=-\frac{2en_{0}}{\epsilon_{0}}\left[{\left(\frac{m_{+}}{2e}\right)}^{\frac{1}{2}}k_{i}n\int_{0}^{z}\frac{dy\;\exp\left[eV\left(y\right)/kT_{e}\right]}{{\left[V\left(y\right)-V\left(z\right)\right]}^{1/2}}-e^{-eV\left(z\right)/kT}\right].$$ (39)

In the Tonks and Langmuir treatment the left-hand side of eq (39) was neglected. From eq (33) we can see that this corresponds to the assumption of charge neutrality or that λ*_D_*_e_/Λ << 1 and results in the neglect of space charge sheaths near the walls.

**Figure 8:**
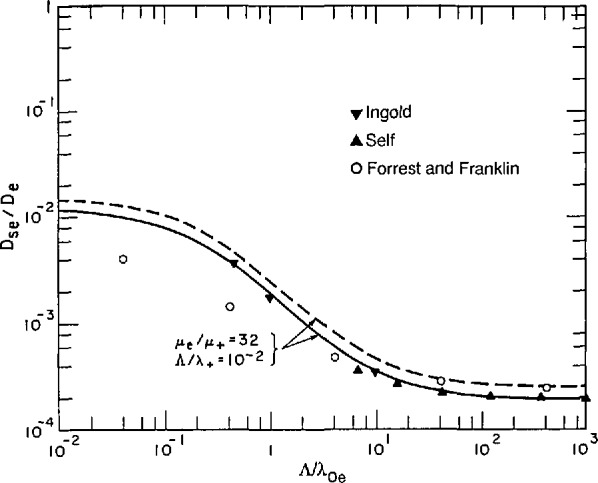
Normalized diffusion coefficients vs Λ/λ_De_ for λ_+_/Λ>> 1 and ions of 1 amu.

Self [49] obtained numerical solutions for the complete eq (39). Once this potential has been obtained one can calculate the flux of charged particles and the effective diffusion coefficient using eqs (35) and (37). The results of this calculation for ions of one atomic mass unit are shown in figure 8. The solid triangles show the results obtained by Self for parallel-plane geometry. The inverted triangles show the results we obtained by extrapolating calculations by Ingold [36] for parallel plane geometry to small values of the Λ/λ_+_ using the relations of section 3.3.5. The open circles show results obtained by Forrest and Franklin [56] for cylindrical geometry. These authors agree with the extrapolated results from Ingold when corrected for geometry at high Λ/λ._De_. Possibly their smaller values of *D*_se_/*D*_e_ at low values of Λ/λ_De_ are the result of a different numerical factor relating the density to the collisionless flow of charge to the wall. Note that the effective diffusion coefficients at large values of Λ/λ_De_ are independent of mass, while at very low Λ/λ_De_ (free fall) the values of *D*_se_/*D*_e_ vary as the square root of the mass. Again the solid curve is an empirical fit to the detailed theory for parallel-plane geometry which will be discussed in section 3.3.5. The dashed curve is calculated using the empirical formula for cylindrical geometry. A simplified model for these conditions, but without explicit consideration of the sheath, has been developed for cylindrical and rectangular geometries by Kino and Shaw [57]. Models developed for application to argon-ion lasers will be cited in section 4.1.

#### 3.3.4 Ambipolar Motion at Various Gas Densities: Λ/λ_De_>>1 and 0 < Λ/λ_+_ < ∞

**Figure 9:**
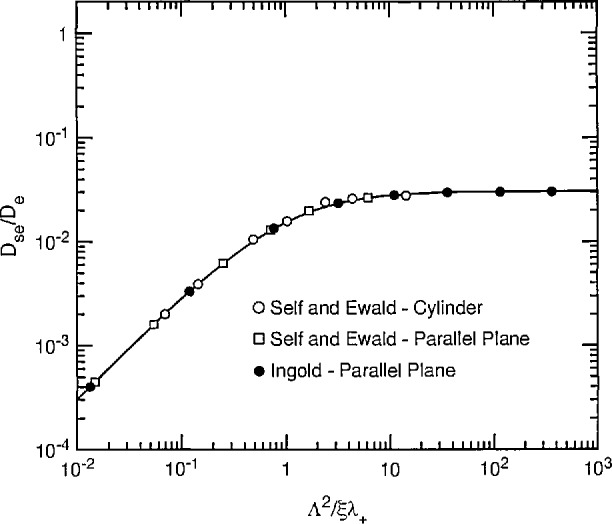
Normalized diffusion coefficients vs Λ^2^/ξλ_+_+ for λ_De_/Λ<<1.

Self and Ewald [58] have combined eqs (35) through (37) with eq (33) to obtain the results shown in figure 9 by the open circles and squares for cylindrical and planar geometry, respectively. The solid circles are results obtained by Ingold [36] for planar geometry. The solid curve is calculated using the empirical formula discussed in section 3.3.5. The results shown in figure 9 correspond to the right-hand side of the square in figure 5. In figure 9 we see that the use of Λ^2^/ξλ_+_ instead of Λ/λ_+_ allows one to compress the calculations for cylindrical and parallel plane geometry into a single curve.

#### 3.3.5 General Solution: 0 < Λ/λ_De_ < ∞ and 0 < Λ/λ_+_ < ∞

**Figure 10:**
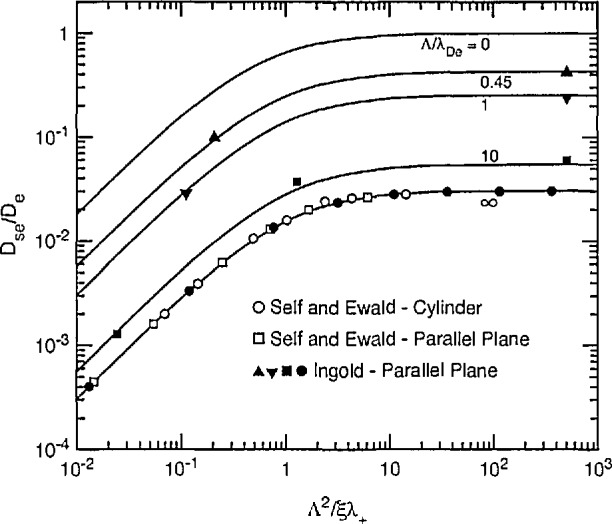
Normalized diffusion coefficients vs Λ^2^/ξλ_+_ for various Λ/λ_De_.

General results appropriate to essentially all of the area of figure 5 have been obtained by Forrest and Franklin [56] and by Ingold [36] for the case of *T*_e_*>>T*_+_, i.e., for the “active” discharge of Allis and Rose [28]. Some of Ingold’s results are shown by the solid points in figure 10. Here again we have used Λ^2^/ξλ_+_ instead of Λ/λ_+_ in order to combine results for cylindrical and parallel plane geometry. The curve shown for Λ/λ_De_ = 0 and the curves and points shown for Λ/λ_De_= ∞ are the same as those in figures 2 and 9, respectively. The curves shown for intermediate values of Λ/λ_De_ are empirical fits obtained by Muller and Phelps [59] by sliding the solid curves for Λ/λ_De_ = 0 downward and slightly to the right, so as to pass through the calculated points from Ingold. In other words, we have assumed that the theory derived for the motion of particles in the absence of space charged fields can be scaled to fit Ingold’s results for various values of the ratio of diffusion length to screening length, including the value of infinity. Extrapolations of these curves to very low values of Λ^2^/ξλ_+_ were used to obtain the points attributed to Ingold in figure 8.

**Figure 11:**
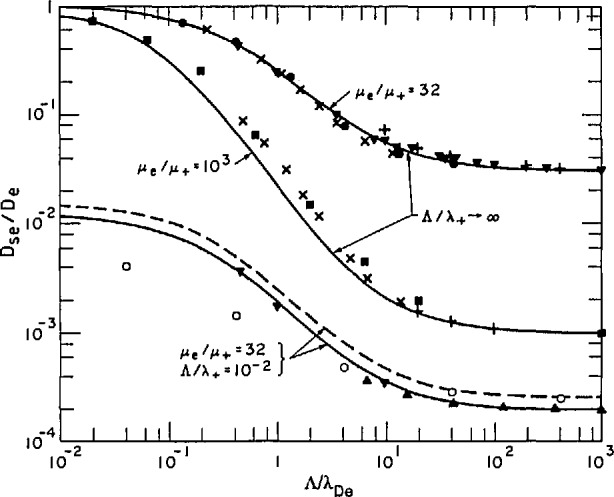
Normalized diffusion coefficients vs Λ/λ_De_= for various Λ/λ_+_ and μ_e_/μ_+_ = *σ* The symbols are the same as in figures 7 and 8.

Ingold’s results [36] were obtained using eq (36) and (37) for the flux of charged particles. He used boundary conditions equivalent to those of section 2.2 for a completely absorbing boundary. In figure 11 we have combined figures 7 and 8 to show the comparison of various calculations as the ratio Λ/λ_De_ is varied. Figures 10 and 11 also show a comparison of the various theoretical calculations of the effective diffusion coefficients with the smooth curves generated by the empirical formula of Muller and Phelps [59]. These formulas have been simplified by Chantry [24] with negligible changes in magnitude. The resulting relation for *D*_se_/*D*_e_ is

$$\frac{D_{\mathrm{se}}}{D_{e}}={\left(1+\frac{\xi \ell_{s}}{\Lambda^{2}}\right)}^{-1}\times \frac{\left(20+10\left(\Lambda /\lambda_{\mathrm{De}}\right)+{\left(\Lambda /\lambda_{\mathrm{De}}\right)}^{2}\right)}{\left(20+12{\left(\sigma +1\right)}^{1/2}\left(\Lambda /\lambda_{\mathrm{De}}\right)+\left(\sigma +1\right){\left(\Lambda /\lambda_{\mathrm{De}}\right)}^{2}\right)},$$ (40)

where the effective linear extrapolation length *ℓ*_s_ defined by Chantry [24] is given by

$$\frac{\ell_{s}}{\lambda_{+}}=\sigma {\left(\frac{\pi m_{e}}{6m_{+}}\right)}^{\frac{1}{2}}\frac{\left(1+3.3\left(\Lambda /\lambda_{\mathrm{De}}\right)\right)}{\left(1+2.34\left(\sigma +1\right){\left[\frac{m_{e}}{m_{+}}\right]}^{\frac{1}{2}}\left[\frac{\Lambda }{\lambda_{\mathrm{De}}}\right]\right)},$$ (41)

σ = μ_e_/μ_+_ and the boundaries are assumed to be completely absorbing. The quantity ξ was discussed in section 2.2. Note that eqs (40) and (41) correct some typographical errors in the original paper [24,60].

In the limit of large electron and ion densities or Λ/Λ_De_>> 1, eqs (40) and (41) reduce to

$$\frac{D_{\mathrm{se}}}{D_{e}}={\left(1+\frac{{\left(\pi /3\right)}^{1/2}\;\xi \;\lambda_{+}\sigma }{\Lambda^{2}\left(1+\sigma \right)}\right)}^{-1}\frac{1}{\left(1+\sigma \right)}.$$ (42)

Alternate empirical expressions derived from the results of Self and Ewald [58] discussed in section 3.3.4 have been given by Ferreira and Ricard [61].

Chantry [60] has suggested that eqs (40) and (41) can be used when *T*_e_ is comparable with *T*_+_, e.g., in thermal equilibrium where *T*_e_ *= T*_+_, by replacing the (1 + σ) factor in these equations by *D*_e_/*D*_a_, where *D*_a_ is given by eq (29).

#### 3.3.6 The Diffusion of Positive Ions

Thus far we have discussed the results in terms of the effective diffusion coefficient for electrons. Since the production rates for electrons and ions by electron impact ionization are equal and the steady-state densities of electrons and ions are unequal, the effective diffusion coefficients for positive ions and electrons must differ. Relatively little effort has been devoted to evaluation of the effective diffusion coefficient for positive ions in a steady-state discharge.

**Figure 12:**
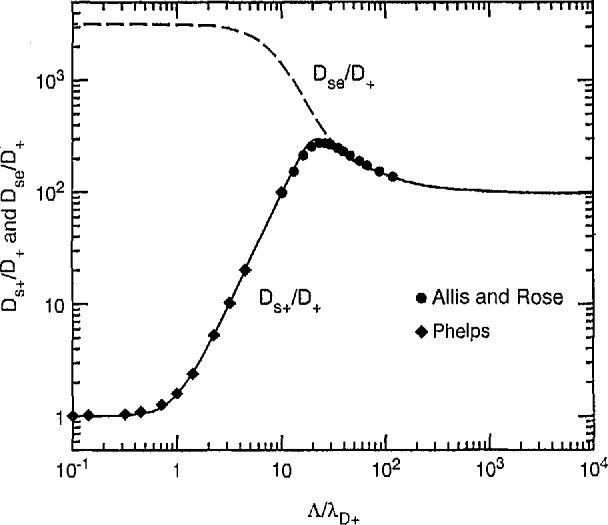
Normalized positive ion diffusion coefficients *D*_s+_*/D_+_* vs Λ/λ*_D_*_+_ for Λ/λ_+_→∞.

The results of Allis and Rose [28] can be used to calculate the effective diffusion coefficient for positive ions at high gas densities. These results are shown by the solid circles in figure 12 where *D*_s+_*/D*_+_ is plotted as a function of Λ/λ*_D_*_+_ for Λ^2^/ξ*ℓ*_+_ ⪢1, where Λ*_D+_* is the screening length for positive ions defined by eq (24) with *q* = +. Values of *D*_se_/*D*_+_ versus Λ/λ*_D_*_+_ are shown by the dashed curve for comparison. This plot is for *σ* = 32 and (*D*_e_/μ_e_)/(*D*_+_/μ_+_) = 100, as one might expect in an active discharge in H_2_. For values of Λ/λ*_D_*_+_ less than about 100 there are significant differences in the effective diffusion coefficients for positive ions and for electrons, but at higher Λ/λ*_D_*_+_ the positive ions and electrons diffuse together as in the am-bipolar limit. The author has extended the results of Allis and Rose to low Λ/λ*_D_*_+_ by assuming that the electron density is small enough so that the electric field is determined only by the ions and that the ion production varies as the electron density, i.e., as cos (*z*/Λ). Thus, self-repulsion dominates the ion motion for 1< Λ/λ*_d_*_+_ < 10. The solid diamonds show numerical results. Numerical and analytical results show that for 2 < Λ/λ*_d_*_+_ < 10, *D*_s+_*/D*_+_ varies as (Λ/λ*_D_*_+_)^2^ For Λ/λ*_D_*_+_ < 1, *D*_s+_/*D_+_* approaches 1 as expected for no space charge effects. The solid curve shows an empirical fit to the data for all Λ/λ_+_. Dote and Shimada [62] have investigated theoretically the diffusion of positive ions in an active discharge, but do not find the maximum in the *D*_s+_/*D*_+_ values found by Allis and Rose [28]. In the afterglow, where *T*_e_ *= T*_+_, Gerber, Gusinow, and Gerardo [63] find a maximum in *D*_s_*_+_*/*D*_+_ similar to that of figure 12. See section 3.5.2.

#### 3.3.7 Summary of Transitional Ambipolar Diffusion In

sections 3.3.1–3.3.6 we have reviewed the available theoretical models describing the loss of charged particles to the wall of the discharge vessel when only electrons and one type of positive ion are present in the gas. The theory for the loss of electrons covers the complete range of ratios of the diffusion length to the screening length and of the diffusion length to the positive ion mean-free path. A relatively simple empirical expression enables one to relate all of the various theoretical results for the diffusion of electrons. The theoretical effective diffusion coefficient for the loss of positive ions to the walls has been extended from that for the ambipolar limit to include all values of Λ/λ*_D_*_+_ for Λ/λ_+_>> 1, but theory is not available for Λ/λ*_D_*_+_ *≈* 1 and λ_+_/Λ *≈* 1.

### 3.4 Electrons and Several Types of Ions

In this section we review work on the simultaneous diffusion of electrons and several types of positive ions and/or negative ions.

#### 3.4.1 Electrons, Positive Ions, and Negative Ions: Λ/λ_De_>> 1 and Λ/λ_+_ (Λ/λ_−_ >> 1

This review of the simultaneous diffusion of electrons, positive ions, and negative ions is largely based on the early treatment of the subject by Oskam [1]. For recent general discussions see Rogoff [64] and Tsendin [65]. The equations appropriate to the simultaneous diffusion of electrons and one type each of positive and negative ions in the limit of Λ/λ_De_>>1 and Λ/λ_+_>>1 are eqs (25) and (26) for electrons and positive ions and

$$\Gamma_{-}\left(z\right)=-D_{-}\;\nabla \;n_{-}-\mu_{-}n_{-}E_{s}$$ (43)

for negative ions. Here Γ_−_ and *n*_−_ are the flux and density of negative ions, while *D*_−_ and μ_−_ are the diffusion and mobility coefficients for the negative ions. As expected, eq (43) for the negative ion current is very similar to eq (25) for the electrons. In the steady state one requires that Γ_e_+Γ_−_ = Γ_+_, so that there will be no accumulation of charge within the discharge. This condition allows one to calculate the space-charge electric field from eqs (25), (26), and (43) as

$$E_{s}=-\frac{\left(D_{e}\nabla n_{e}+D_{-}\nabla n_{-}-D_{+}\nabla n_{+}\right)}{\left(\mu_{e}n_{e}+\mu_{-}n_{-}+\mu_{+}n_{+}\right)}.$$ (44)

This rather complicated expression simplifies when we take into account the condition that Λ/λ_De_>> 1 so that there is charge neutrality, i.e., the sum of the electron and negative ion densities equals the positive ion density. Further simplifications result if we follow Seeliger [66] and Oskam [1] and assume that the spatial distributions of electrons and all ions are the same, i.e., that

$$\frac{\nabla n_{e}}{n_{e}}=\frac{\nabla n_{-}}{n_{-}}=\frac{\nabla n_{+}}{n_{+}}\equiv \frac{\nabla n}{n},$$ (45)

and that *n*_−_/*n*_e_ = α. When the congruence assumption represented by eq (45) is valid, the electric field is then given by

$$E_{s}=-\frac{\left(D_{e}+\alpha D_{-}-\left(1+\alpha \right)D_{+}\right)}{\left(\mu_{e}+\alpha \mu_{-}+\left(1+\alpha \right)\mu_{+}\right)}\frac{\nabla n}{n}.$$ (46)

Since the diffusion coefficient and mobility coefficient for electrons are much larger than the corresponding coefficients for negative ions and positive ions, this expression becomes rather simple when αμ_−_ and αμ_+_ << μ_e_, i.e., when the value of α is less than about 10. In this case the space charge electric field is determined by the electron s just as in the absence of negative ions, i.e., as in the second form of eq (27). The ambipolar diffusion coefficients for this situation are given by

Dae=(1+α)D+(1+TeT)+αD−(TeT−1),→((1+α)μ++αμ−)Deμe, (47)

$$\begin{array}{l} D_{a-}=\left[2\left(1+\alpha \right)\frac{D_{+}}{D_{e}}-\frac{T_{e}}{T_{-}}+1\right]D_{-}\\ \;\to -\frac{D_{e}}{\mu_{e}}\mu_{-}, \end{array}$$ (48)

and

$$D_{a+}=\left(1+\frac{T_{e}}{T_{+}}\right)D_{+}\to \frac{D_{e}}{\mu_{e}}\mu_{+}.$$ (49)

Here we have used the relationship *kT_i_*/*e = D*_i_/μ_i_ to simplify the equations. The second forms of the equations are the limiting forms for *T*_e_*>>T_−_* and *T*_+_. Of particular interest is the negative value for *D*_a−_, which means that the negative ions flow toward the center of the discharge. This phenomenon leads to a breakdown of the assumption of congruent spatial distributions of the ions and electrons and eventually to a contraction of the discharge [66–72]. The consequences of this flow are discussed below. When *T*_e_ *= T_−_ = T_+_*, then *D*_ae_ = (1 + *α*) 2*D +*, *D*_a−_ = (1 + α) 2*D_+_D_−_/D*_e_, and *D_a+_*= 2*D*_+_. These results would be appropriate to an afterglow where the electrons have cooled to the gas temperature. See section 3.5.1.

The equations of continuity for the electrons, negative ions, and positive ions are

$$\frac{\partial n_{e}}{\partial t}=-\nabla \cdot \Gamma_{e}+k_{i}\;n\;n_{e}-k_{a}\;n\;n_{e}+k_{d}\;n\;n_{-},$$ (50)

$$\frac{\partial n_{-}}{\partial t}=-\nabla \cdot \Gamma_{-}+k_{a}\;n\;n_{e}-k_{d}\;n\;n_{-},$$ (51)

and

$$\frac{\partial n_{+}}{\partial t}=-\nabla \cdot \Gamma_{+}+k_{i}\;n\;n_{e}.$$ (52)

Here *k*_a_ is the electron attachment rate coefficient, *k*_d_ is the collisional detachment rate coefficient for the negative ions, and *k*_i_ is the ionization rate coefficient. In eq (50) we have indicated that the electrons may be lost by flow and by electron attachment, while they are produced by electron impact ionization and by collisional detachment from the negative ion. We have neglected potentially important processes such as electron-positive ion and positive ion-negative ion recombination. In eq (51) we see that in this model the negative ions are lost by flow and by collisional detachment, and are produced by electron attachment to the neutrals. Finally, eq (52) gives the positive ion loss by flow and production by electron impact ionization. We are particularly interested in the steady state equation for the negative ions.

If we solve eqs (50)–(52) for the steady-state ratio of the negative ion density to the electron density a, we obtain

$$\alpha =\frac{n_{-}}{n_{e}}=\frac{k_{a}\;n}{\left(k_{d}\;n+D_{a-}/\Lambda^{2}\right)}\to \frac{k_{a}\;n}{\left(k_{d}\;n-\left(D_{e}/\mu_{e}\right)\left(\mu_{-}/\Lambda^{2}\right)\right)}.$$ (53)

The second form of eq (53) applies when *T*_e_/*T*_−_ >> 1 and shows that when the diffusion contribution is less than collisional detachment, the value of *α* is positive as required for a meaningful solution [65]. In this case one pictures the negative ions as diffusing toward the center of the discharge tube where they undergo collisional detachment. The negative charge then diffuses toward the wall of the discharge tube in the form of electrons. In a real plasma one has to be concerned that the assumption of the same spatial distribution for all ions may not be valid [65,73].

The consequences of various limiting values of *α* are discussed further by Oskam [1] and Rogoff [64]. The case of very large *α* leads to almost free diffusion of the electrons in the presence of large densities of positive and negative ions. This case will be discussed further in connection with afterglow models and experiment in section 3.5.1.

The effects of the change in steady-state, space charge fields produced by negative ions have been considered for flowing afterglows [74] and high pressure mass spectrometers [75].

#### 3.4.2 Electrons and Several Types of Positive Ions

We now consider the situation with several types of positive ions and no negative ions [1,64,76–78]. The particle flux equations for electrons and two types of positive ions, labeled 1 and 2, are

$$\Gamma_{e}\left(z\right)=-D_{e}\;\nabla \;n_{e}-\mu_{e}\;n_{e}\;E_{s},$$ (25)

$$\Gamma_{1}\left(z\right)=-D_{1}\;\nabla \;n_{1}+\mu_{1}\;n_{1}\;E_{s},$$ (54a)

and

$$\Gamma_{2}\left(z\right)=-D_{2}\;\nabla \;n_{2}+\mu_{2}\;n_{2}\;E_{s}.$$ (54b)

Here the Γ*_j_* and n*_j_* are the fluxes and densities of ions 1 and 2 and the *D_j_* and μ*_j_* are the diffusion coefficients and mobilities for these ions. In the steady-state Γ_e_ = Γ_1_ + Γ_2_ so that

$$E_{s}=-\frac{\left(D_{e}\nabla n_{e}-D_{1}\nabla n_{1}-D_{2}\nabla n_{2}\right)}{\left(\mu_{e}n_{e}+\mu_{-}n_{-}+\mu_{+}n_{+}\right)}\to -\frac{D_{e}}{\mu_{e}\;}\frac{\nabla n}{n}.$$ (55)

This equation is very similar to eq (44). If one makes the assumptions that the ratio of ∇*n*/*n* is the same for each ion and for the electrons, as in eq (45), and that the plasma is quasineutral, *n*_1_ + *n*_2_ −*n*_e_<<*n*_e_, one obtains the familiar expression for the electric field given in the second form of eq (55). Substitution of this expression into eqs (25), (54a), and (54b) yields expressions for the ambipolar diffusion coefficients for each of the ions and for the electrons. These relationships were used by Phelps [77] to analyze the observed decay of helium ions and of electrons in the afterglow of a helium discharge. Note that the assumption of congruence, i.e., the equality of the ∇*n*/*n* values for the electrons and both types of ions, is not generally applicable [1,78]. It should apply when nonlinear loss and production processes, such as electron-positive ion and positive ion-negative ion recombination, are negligible and the ions are distributed in the fundamental diffusion mode [1,77]. In the case of positive ions only, we do not have the tendency for diffusion to destroy congruency as we did in the negative ion case of section 3.4.1.

### 3.5 Ambipolar Diffusion in Afterglow

Many experimental measurements and theoretical analyses have been applied to the diffusion of charged particles in the afterglow of a discharge. See Oskam [1] for an extensive review of the subject. Here we will be concerned with the afterglows which are dominated by diffusion. Reactions which are nonlinear in the charged particle densities, such as recombination, are neglected. The diffusion models discussed previously for steady-state electrical discharges are applicable to many afterglow experiments. We will also be concerned with departures from the description which have been given in previous sections, i.e., with phenomena such as diffusion cooling.

#### 3.5.1 Isothermal Plasmas

In this section we assume that the electron temperature is equal to the positive ion temperature and that both of these temperatures are equal to the gas temperature, i.e., *T*_e_ *= T_+_ = T*_g_. We also assume that λ_De_/Λ << 1 and λ+/Λ << 1. For these conditions we obtain the by now familiar expression for electron particle flux in terms of the ambipolar diffusion coefficient given by eq (29). When this equation is substituted into the time-dependent electron continuity equation, we obtain

$$\frac{\partial n_{e}}{\partial t}=D_{a}\nabla^{2}n_{e},$$ (56)

where *D*_a_ is given by eq (29). We consider the solution to eq (56) for sufficiently long times such that higher order diffusion modes have disappeared. The conditions necessary for the neglect of higher order diffusion modes are discussed in detail by McDaniel [5]. The result for the lowest diffusion mode is

$$n_{e}\left(t\right)=n_{e}\left(0\right)e^{-t/\gamma },$$ (57)

where

$$\frac{1}{\gamma }=\frac{D_{a}}{\Lambda^{2}}=\frac{2D_{+}}{\Lambda^{2}}=\frac{2kT_{+}}{e\Lambda^{2}}\mu_{+}.$$ (58)

The final form of eq (58) is often used to obtain the ion mobility from measurements of the decay constant for the electron and ion densities [1,5,79]. Because of various ion conversion processes [1,77], not included in eqs (56)–(58), it is generally necessary to plot the experimental values of *n*/*γ* as a function of *n* and extrapolate to *n* = 0. Many authors have used this technique to determine ion mobility and under proper circumstances it can yield reliable information. However, under many circumstances it has not yielded ion mobilities that agree with those measured with drift tube techniques [6,79]. We therefore need to consider sources of error in the interpretation of afterglow experiments [80]. First, we will consider the departures from ambipolar diffusion and the solutions given by eqs (57) and (58) which occur when $\lambda_{D_{e}}$ becomes comparable with A. Second, we will discuss the phenomenon of diffusion cooling.

#### 3.5.2 Departures from the Ambipolar Limit

**Figure 13:**
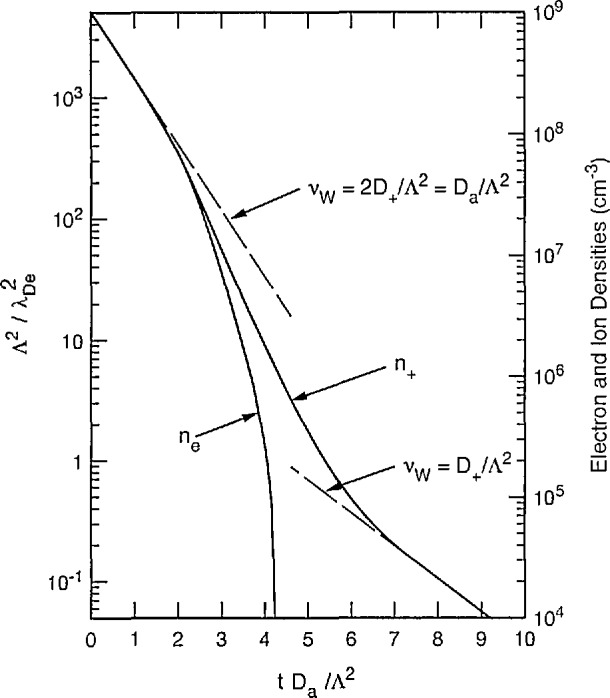
Schematic of afterglow transients showing transitional ambipolar diffusion.

Measurements and models of the decay of electron density and ion wall current in the afterglow of pulsed discharges in helium give very direct evidence of the transition from ambipolar to free diffusion [41,63,81–84]. Figure 13 shows schematically the results of numerical solutions by Gusinow and Gerber [82] of eqs (25), (26), (32), and (33) and of eqs (1) with *k*_i_ = 0 for the time-dependence of electron and ion densities in a helium afterglow. At early times the electron and ion densities are equal and decay with a reciprocal time constant of *DJ* Λ^2^ = 2*D*_+_/Λ^2^. When the charge density decreases such that when $\Lambda /\lambda_{D_{e}}\approx 10$ there is a more rapid decay of the electron and ion densities. At still lower values of $\Lambda /\lambda_{D_{e}}$ the electron and ion densities no longer decay together. The electron decay continues to become more rapid until it approaches the free diffusion rate. The positive ions, on the other hand, soon reach a maximum decay rate corresponding to the peak in figure 12. Their decay rate then decreases to a value characteristic of the free diffusion of the positive ions, i.e., 1/*τ* = *d*_+_/λ^2^. During this transition the ambipolar field decreases to zero. This transition has also been analyzed [85] for infinite spherical geometry using the constant ion density ratio approximation discussed in section 3.3.5.

In the case of afterglows in which negative ions are present the electrons disappear rapidly when Λ/λ_De_ ≈ 1 and one is left with a plasma composed of negative and positive ions. According to eq (46) there is very little space charge electric field. A number of investigators have shown experimentally [86–88] and theoretically [41] that since the electric field is no longer sufficient to stop the negative ion flow, the negative ion wall current suddenly increases at this time. Qualitatively similar results have been obtained [89] using the constant ratio ion density approximation.

An interesting feature of these theories and experiment is the apparent applicability of the steady-state results to experimental afterglows [81]. This effect is presumably the result of the ability of the highly mobile electrons to readjust their density rapidly on the time scale of interest.

#### 3.5.3 Diffusion Cooling

A second phenomenon, which can cause significant errors in the simple model of the diffusion of ions in an afterglow, is that of diffusion cooling. This effect was discovered by Biondi [90] in neon afterglows where he observed that the apparent diffusion coefficient for the electrons and ions dropped by almost a factor of two at low gas densities. This effect is particularly pronounced in neon because the relatively small ratio of electron to atom mass and low momentum transfer cross section for electrons in neon result in poor energy exchange between electrons and gas atoms. Biondi showed that the addition of small amounts of helium restored the thermal contact between electrons and the gas and led to higher values for the effective diffusion coefficient.

**Figure 14:**
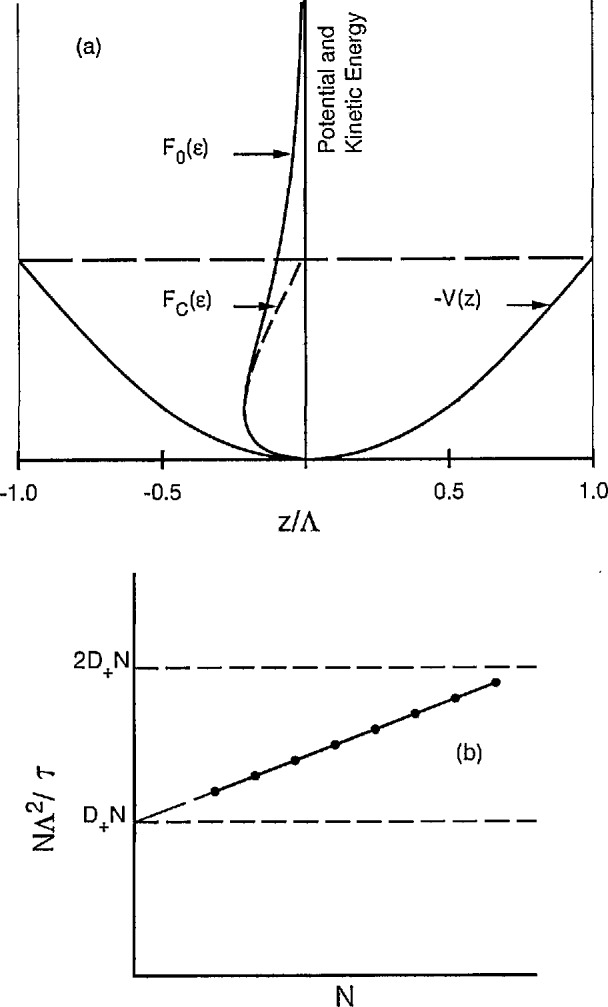
Schematic of electron energy distributions and data in diffusion cooling experiment.

Figure 14(a) shows in a schematic fashion the potential well and electron distribution functions appropriate to the diffusion cooling problem. The potential well has the typical parabolic shape at the center and reaches the finite value at the walls of the container. Electrons with energies larger than that indicated by the horizontal dashed line can escape from the well to the walls. If the frequency of energy relaxation collisions is sufficiently rapid, only those electrons that are very close to the wall will be able to cross the potential barrier and the space charge field in most of the plasma will be unperturbed from the ambipolar value. As the energy relaxation frequency is reduced by decreasing the elastic collision frequency or by increasing the mass of the atom, electrons from near the center of the container can reach the wall without undergoing energy relaxation. The effect of this process is to deplete the high energy tail of the distribution as indicated by the unperturbed energy distribution *F*_0_(*ϵ*) shown by the solid curve and the cooled distribution *F*_0_(*ϵ*) shown by the dashed curve. The loss of high energy electrons reduces *D_e_*/μ_e_, i.e., reduces the effective temperature of the electrons, and reduces the space charge electric field and the loss of ions by ambipolar diffusion. In the limit of *D*_e_/μ_e_→0, the ambipolar field goes to zero and the ions diffuse freely. Detailed theoretical treatments of diffusion cooling are now available [91–94].

Figure 14(b) shows the schematic of the values of *n*Λ^2^/*τ* as a function of gas density under conditions in which diffusion cooling is important. The points and line through them indicate qualitatively the kind of experimental diffusion coefficient results obtained [88,90]. Diffusion cooling has also been observed via measurements of the decrease in radiation temperature of the electrons [95,96]. Unfortunately, the density dependence of the apparent ambipolar diffusion coefficient caused by diffusion cooling is qualitatively similar to that which would be expected if the ions were being converted from an ionic species with a low diffusion coefficient to a species with a high diffusion coefficient [77]. This possibility illustrates the need for mass spectrometric identification of the ions in such experiments.

We note that the phenomenon of electron motion from one portion of the plasma to another in times which are fast compared to energy relaxation times, which is responsible for diffusion cooling, is also responsible for excess ionization in the center of the discharge in the steady-state “active” discharge to be discussed in section 4.1.2.

## 4. Applications of Models of Ambipolar Diffusion

We now turn to applications of the models of ambipolar diffusion discussed in sections 2 and 3 to the interpretations of several gas discharge systems. Firstly, we consider the maintenance of steady-state dc and microwave discharges at low, moderate, and high pressures. Secondly, we summarize the effects of magnetic fields on the diffusive loss of charged particles from discharges. We then briefly discuss the role of diffusion in transient discharges at high pressures.

### 4.1 Steady-State Discharge Maintenance

In this section we apply the models developed in sections 2 and 3 to the prediction of the applied electric fields, gas densities, etc. required to maintain a low power, low pressure electrical discharge in which the ionization is by single-step electron impact excitation of ground state atoms or molecules. In most models of steady-state discharges the ambipolar electric fields *E*_s_ are perpendicular to the applied electric field *E*_a_ and the effects of the ambipolar fields on the electron energy distribution function, the excitation and ionization rate coefficients, and transport in the direction of the applied field are neglected. In section 4.1.2 we will, however, summarize work on the effects of ambipolar electric fields on the electron energy distribution functions and the resultant changes in applied fields necessary to maintain the discharge.

#### 4.1.1 Discharge Maintenance at Moderate Gas Densities: (Λ/λ_+_ >> 1, Λ/λ_u_ >> 1, and 0 < Λ/λ_De_ < ∞)

We now consider the application of the theory developed for the effective diffusion coefficient for electrons to the calculations of the properties of an electric discharge. Equation (34) shows the balance between the production of electrons by electron impact ionization at the axis of the discharge and the loss of electrons by diffusion. This equation can be written as

$$n^{2}\Lambda^{2}=\frac{D_{\mathrm{se}}}{D_{e}}\frac{\left(D_{e}n\right)}{k_{i}}.$$ (59)

Rose and Brown [97] have applied eq (59) and the theory developed in section 3.3.5 to an analysis of steady-state microwave discharges in H_2_ and find generally good agreement between theory and experiment. Poor agreement with the simple theory represented by eq (59) is found for Ar at microwave frequencies and at dc, presumably because cumulative or multistep ionization processes are important for the rare gases [98]. Muller and Phelps [59] have applied the results discussed in section 3.3.5 for *D*_se_/*D*_e_ to an analysis of low current discharges in H_2_-He mixtures and find good agreement with experiment at their higher gas densities. Hydrogen and H_2_-He gas mixtures are suitable for these comparisons because of the short lifetime of metastable H_2_ states [99] and rapid quenching of the He metastables through Penning ionization of the H_2_ [4].

In the analysis of the H_2_-He positive column discharge, eq (59) has been used to calculate the product nA using theoretical values of the ratio *D*_se_/*D*_e_ and of *nD*_e_/*k*_i_. The *D*_se_*/D*_e_ values are given by the empirical expressions in eqs (40) and (41) involving the ratios *λ*_De_/Λ, λ_+_/Λ, and ξ/Λ. The ratios λ_De_/Λ and λ*_+_*/Λ are calculated using the equations

$$\frac{\lambda_{\mathrm{De}}^{2}}{\Lambda^{2}}=\frac{eD_{e}/\mu_{e}}{8\pi \;Ry\;a_{0}}\frac{e\;c\;A\;W_{e}}{I\;\Lambda^{2}},$$ (60)

and

$$\frac{\lambda_{+}^{2}}{\Lambda^{2}}={\left(\frac{m_{+}\mu_{+}n}{e}\right)}^{2}\frac{3\;e\;D_{e}}{m_{+}\;\mu_{e}}\frac{1}{n^{2}\;\Lambda^{2}},$$ (61)

where *c* is the ratio of the mean electron density to its peak value and *A* is the area of the discharge. Equation (60) is a reformulation of eq (24). Note that the second factor in eq (60) is the electron density on the axis of the discharge. For a long cylindrical discharge the radius cancels out of eq (60) and the ratio Λ^2^/λ_De_^2^ is proportional to *I*. Keeping in mind that *nD*_e_, *D*_e_/μ_e_, and *k*_i_ are functions of *E*_a_/*n*, one sees that eqs (59)–(61) also show the applicability of the experimental scaling parameters [4] *n*Λ, *J/n*^2^, and *E*_a_/*n*, where *J ≡ I/A* is the current density at the axis of the discharge. The definition of the mean-free path of the positive ions in eq (61) is that given by Ingold [36] and involves the ion mass *M_+_*, the ion mobility μ*_+_*, and the *D*_e_*\*μ_e_ value for electrons. The first factor on the right hand side of eq (61) is the square of the mean-free-time for ion-neutral collisions per atom evaluated from the ion mobility. The ion speed is evaluated at the effective temperature *D*_e_*\*μ_e_ for the electrons and is determined from theory or experiment by the *E*_a_/*n* value for the given gas or gas mixture.

**Figure 15:**
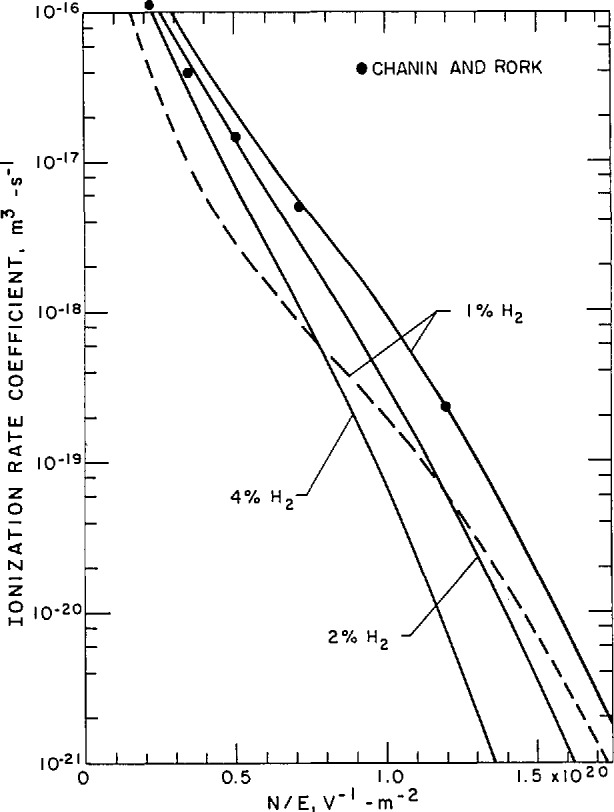
Ionization coefficients for H_2_-He mixtures.

Figure 15 shows the results of calculations of the ionization rate coefficient *k*_i_ as a function of *E*_a_/*n* for the mixture of helium and hydrogen that was used in the experiments. These curves were calculated using electron collision cross section sets for helium and hydrogen and taking into account the Penning ionization of the hydrogen. The characteristic electron energy *E*_a_/*n* is a relatively slowly varying function of *E*_a_/*n* and is not shown.

**Figure 16:**
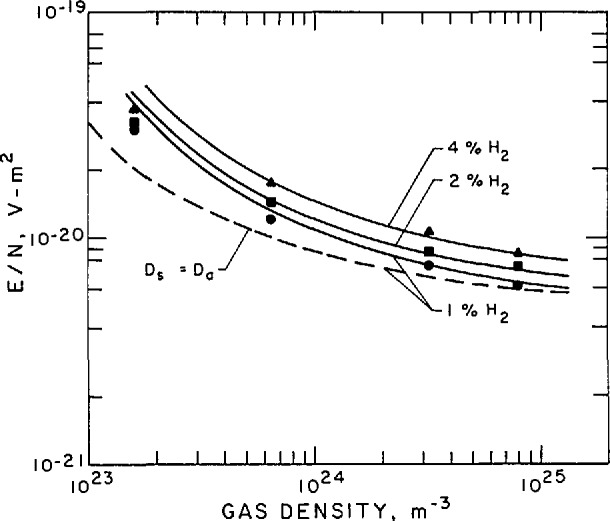
Maintenance *E/n* for H_2_−He mixtures showing comparison of experiment with predictions of transitional ambipolar diffusion theory.

Figure 16 shows the results of this analysis. In this figure the *E*_a_/*n* values are plotted as a function of *n* for several different fractional concentrations of hydrogen in helium. The points are the experimental data and the smooth curves are the results of calculations. The dashed curve shows values of *E*_a_/*n* predicted when the diffusion loss is assumed to be given by the ambipolar limit. The solid curves show the results when departures from ambipolar diffusion are taken into account. The solid curves show good agreement with experiment at the higher values of *n* Λ. At the lower values of *n*Λ there is small but systematic disagreement between theory and experiment. The sign of this disagreement suggests that the theory has omitted a source of ionization. Muller and Phelps [59] propose that the source of ionization is the motion of the electrons from the outer portion of the discharge toward the axis in the space charge potential as discussed in section 4.1.2.

Many other comparisons have been made between positive column models and experiment. In most of them the model is very complicated because of the important ionization resulting from electron-metastable and metastable-metastable collisions [62,100–102]. Here we have cited only some of the more recent papers.

#### 4.1.2 Radial Nonequilibrium at Low Gas Densities: (Λ/λ _+_ >> 1, Λ/λ_u_⩽1, and 0 < Λ/λ_De_< ∞)

In this section we are concerned with the changes in the ionization rate coefficient *k*_i_ resulting from the motion of the electrons in the potential well created by the space charge electric field as compared to the ionization coefficient in the absence of such a field. This problem has been addressed for microwave discharges by Bernstein and Holstein [103] and for dc discharges by Blank [104], Herrmann, Rutscher, and Pfau [105], and by Tsendin [106,107]. Radial energy nonequilibrium effects are very important in the low-pressure discharges used for rare gas, ion lasers [108–111].

The origin of the excess ionization is illustrated by considering electrons at large radii of the discharge which are accelerated in the axial direction to kinetic energies just below the ionization potential and then move radially inward at constant total energy to reach kinetic energies above the ionization potential. These electrons are less likely to suffer inelastic collisions than those accelerated to the same final kinetic energy in a spatailly uniform electric field. After producing ionization these electrons may move radially outward at low kinetic energies where, at least in the rare gases, energy losses in inelastic collisions are small. In some models [112] the spatial change in the electron energy distribution function is approximated by a radially varying electron temperature. This process is one of “diffusion heating” and, in a sense, is the inverse of the diffusion cooling discussed in section 3.5.3.

Thus far, there appear to be no simple expressions that allow estimates of the magnitude of the increase in ionization resulting from this nonequilibrium effect.

The nonequilibrium effects caused by radial am-bipolar fields discussed in this section and in section 3.5.3 do not require the introduction of a third dimension for the map of figure 5. The importance of nonequilibrium is determined by the ratio Λ/λ_u_, where λ_u_ is an energy relaxation distance for electrons. We define λ_u_ in terms of the energy exchange collision frequency *ν*_u_/n used in some analyses of electron transport data [113]. Thus, for *D*_e_/μ_e_>>*kT*_g_,

$$\lambda_{u}=\frac{1}{\nu_{u}}{\left(\frac{2e\;D_{e}}{m_{e}\;\mu_{e}}\right)}^{\frac{1}{2}}=\frac{n}{W_{e}\;E}{\left(\frac{2\;\mu_{e}}{e\;m\;D_{e}}\right)}^{\frac{1}{2}},$$ (62)

where *W*_e_ is the electron drift velocity at the discharge *E*_a_/*n*. Note that this relaxation distance is a property of the whole of the electron energy distribution and so may be only a rough measure of energy relaxation for the high energy tail of the electron energy distribution which is of importance in the nonequilibrium ionization. Since the ratio λ_u_/λ_+_ is a constant for a given gas and *E*_a_*/n*, the overall scaling discussed in section 3.3.1 should be preserved.

Measurements of radial ambipolar electric fields *E*_s_ in active discharges have been made by Baghuis et al. [114] and by Ganguly and Garscadden [115]. In the first case the data were obtained at rather high pressures and currents, i.e., Λ/λ_+_>>1, Λ/λ_u_>> 1, and Λ/λ_De_>> 1, and the agreement with predictions [101] is good. In the latter case no comparison with discharge models was made.

Note that ion energy relaxation effects have already been included in the models of section 3.

#### 4.1.3 Diffusive Nonequilibrium in High Pressure Arcs

The phenomenon of diffusive nonequilibrium in high pressure arcs leads to effects such as diffusive separation or demixing of components of the gas [116, 117]. Ambipolar diffusion also plays an important role in departures from local thermodynamic equilibrium by depleting the ions through transport to the wall where they recombine with electrons to produce neutrals which flow toward the center of the discharge [118–122]. Because the analysis of such effects involves the competition between diffusion, ionization, and the nonlinear loss process of electron-ion recombination, we will not consider the models used to describe these effects. Note that we have cited only some of the more recent references.

### 4.2 Magnetic Field Effects

The research on the effects of magnetic fields on space charge dominated plasmas is much too extensive to summarize in this paper. Therefore, we limit the discussion to applied electric fields parallel to the magnetic field, as in the case of magnetic field lines parallel to the axis of the positive column of a glow discharge; to quiescent ranges of plasma parameters; to weakly ionized plasmas; to no relative motion of the magnetic field and the neutral gas; and to the ambipolar limit of λ_De_/Λ<< 1 [123–127]. The basic relations governing the transport of a single type of charged particles in the presence of a magnetic field are given by a number of authors [7,127] and are often expressed as modifications of the diffusion and mobility coefficients. For energy independent collision frequencies for electrons *ν*_em_ and ions *ν*_in_ with the gas, the coefficients describing electron transport parallel to the electric field and transverse to the magnetic field are:

$$\mu_{\mathrm{eT}}=\frac{\mu_{e}}{1+\omega_{Be}^{2}\nu_{\mathrm{em}}^{-2}},\;D_{\mathrm{eT}}=\frac{D_{e}}{1+\omega_{Be}^{2}\nu_{\mathrm{em}}^{-2}}.$$ (63)

In addition, the electron transport transverse to the magnetic field but perpendicular to the electric field is characterized by

$$\mu_{\mathrm{ep}}=\frac{\mu_{e}\;\omega_{eB}\;\nu_{\mathrm{em}}}{1+\omega_{Be}^{2}\;\nu_{\mathrm{em}}^{-2}},$$ (64)

while transport along the magnetic field is independent of *B*. Here *ω_Be_ ≡ eB/m*_e_, where *B* is the magnetic field. Alternate derivations [124, 128] of eqs (63) and (64) show that one can replace the *ων* products by the corresponding values of *ß*_e_ = *μ*_e_*B = ω_Be_*/*ν*_em_. The proper averaging of these expressions for gases in which the collision frequency varies with electron energy has been discussed by various authors [7, 127]. In the limit of low *B/n* the v’s are much larger than the ω’s and one recovers the *D*_e_ and μ_e_ values in the absence of a magnetic field. The corresponding equations for ions are obtained by replacing *ω_b__e_/ν*_em_ by *ω_b_*/*ν*_im_ = μ_i_B ≡ *β*_i_, where *ω_B_*_i_*≡ eB/M* and *M* is the ion mass. The details of averaging of the mobility equations for ions have been discussed by Shunk and Walker [129].

Because of the ease with which a magnetic field prevents the transport of electrons across field lines relative to their transport along the field lines, the predictions of the models of ambipolar diffusion in the presence of a magnetic field are very dependent on the geometry [123–132]. The models usually assume spatially uniform electron and ion temperatures, a single type of positive ion, and negligible electron-ion collisions. We first consider a steady-state plasma in which the net flow of charge to any surface element is zero, as for a container with nonconducting surfaces, and for which $\beta_{e}^{2}>>1$and *β*_i_ is of the order of unity. In this case the effective ambipolar diffusion coefficient *D*_a_*_B_* is given by [124, 125, 131, 132]:

$$D_{aB}\to \frac{D_{a}}{1+\mu_{e}\mu_{i}B^{2}}.$$ (65)

On the other hand, when the surfaces (or a second plasma) at the “end” of the plasma or the use of a metallic container allow efficient transport of charge across magnetic field lines, the effective diffusion coefficient is given by [123, 126, 130, 133]

$$D_{aB}\to \frac{D_{a}}{1+\mu_{i}^{2}B^{2}}\;\mathrm{for}\;\beta_{e}>\;>1.$$ (66)

Here the electrons move along the magnetic field lines until they reach the conducting end plates where they move radially. The length of the plasma in the direction of the magnetic field is assumed large enough so that ion loss in the direction of *B* is still small compared to that in the radial direction. The effects of sheaths at the conducting surfaces are generally neglected.

The quantitative experimental verification of eqs (65) and (66) in active discharges appears to be lacking. Not only is it difficult to satisfy the conditions of either an insulating or a highly conducting “end plate,” but other assumptions of the model are often not met. For conducting end walls, Simon [123,130] found agreement with the magnetic field dependence of eq (66), but found only rough agreement of the magnitudes of the diffusion rates with his experiments and the early results of Bohm et al. [134]. Experimental results with varying degrees of insulating walls have shown significant discrepancies with theory. For example, in several experiments with positive column discharges the apparent transverse diffusion coefficient initially increases with increasing magnetic field and then begins to decrease approximately as predicted by eq (65) [112, 135, 136]. One reason for this effect is that at zero magnetic field and at low enough gas densities λ_u_/Λ is large enough such that the electron energy distribution and “temperature” vary significantly with radius as discussed in section 4.1.2. When an axial magnetic field is raised such that *β*_e_≽ 1 the effective energy relaxation length and the radial transport of electron energy are significantly decreased, the average ionization rate coefficient is reduced, and the axial *E*_a_/*n* required to maintain the discharge increases. Models of the first maximum in *E*_a_/*n* have also included the effects of metastables [136]. The role of ionization waves has been debated [112,137]. A second and more dramatic increase in the apparent transverse diffusion coefficient above the values predicted by eqs (65) and (66) at *β*_i_, values greater than about unity is attributed to the onset of plasma instabilities [12,135,138,139]. The discussion of this effect is beyond the scope of this paper.

An interesting and apparently unanswered experimental question is whether the radial electric field *E*_s_ reverses sign as predicted [124] when eqs (63) and the corresponding equations for ions are substituted into eq (27) and when the magnetic field is such that *D*_eT_ *= D*_iT_. Similar predictions have been made for the λ_+_/Λ >>1 case [140]. There does not appear to be agreement as to whether conducting end walls are sufficient to short circuit the plasma [123, 130] or whether a conducting outer cylinder is needed to avoid electron emission problems at the walls [141]. When electron emission is required to return electrons to the plasma, a significant “cathode fall” voltage could occur at the end plate.

The theory of section 3.3.4 has been extended to treat the diffusion of electrons and ions in a partially ionized gas subject to a magnetic field for λ_De_/Λ<<1 and variable λ*_+_*/Λ [140–143]. There appear to be no quantitative comparisons of these models with experiment [144].

Afterglow plasmas have also been used in attempts to verify the effects of a magnetic field on ambipolar diffusion [125]. Here the comparison should be simpler because of the direct measurement of the deionization rate and the possibility of thermalizing the electrons through collisions with the gas so as to achieve equal electron and ion temperatures. However, there is considerable difference among authors. Some experiments [145] show quantitative agreement with theory for magnetic fields below the onset of instabilities, while others [146–148] find varying degrees of agreement for containers with end walls of unknown effective conductivity. Theory [149] and experiments [150] show that the decay rate is highly sensitive to the alignment of the discharge tube with the magnetic field.

### 4.3 Transient Discharges

The onset of ambipolar diffusion plays an important role in the development of many pulsed discharges by limiting the diffusive loss of electrons and leading to the development of a constricted, highly ionized region or channel. Here we will cite only a limited number of examples.

Ambipolar diffusion is usually included or assumed in models of the development of highly conducting channels or sparks at moderate over-voltages [16, 151, 152]. The reduced loss of electrons due to space charge fields allows the build up of ionization to values that result in the onset of processes such as multistep ionization, electron detachment, and thermal gas expansion.

The effects of radial electric fields caused by diffusive separation of charge appear to be small for the fast time scale and high voltages associated with the growth of the “streamer” which occurs in the later stages of electrical breakdown at high overvoltages in initially uniform and nonuniform electric fields [153].

Ambipolar diffusion has been suggested by Van Brunt and Kulkarni [154] to be important in determining the minimum time between negative corona pulses. However, details of the calculations [155] have not been reported.

One of the mechanisms leading to an increased growth of ionization in the later stages of laser breakdown is the reduction of electron loss by diffusion when the charged particle densities become large enough so that the screening length is comparable with the dimension of the region illuminated by the laser [156].

## 5. Discussion and Summary

We have reviewed the models that have been developed to describe measurements of the loss of electrons and ions by diffusion from weakly ionized gas discharges or plasmas to the walls of a discharge vessel. Scaling parameters for the models discussed and for a given gas are λ_+_/Λ, λ_De_/Λ, E_a_λ_e_, and *ω_B_*/*ν*_in_ when the plasma is subject to a dc applied electric field. Of course, any combination of these parameters leading to the same total number of these parameters is also acceptable. When the discharge is excited by an ac electric field the parameter *ω/ν*_i_ must also be included. These model parameters translate into the experimental parameters of nΛ, *J/n*^2^, *E*_a_*n*, and *B/n* for the dc case in infinite cylindrical or parallel plane geometry, with *ω/n* added for the ac case. In the absence of a magnetic field and at sufficiently high rf fields the models are rather complete and have been tested against experiment. The predictions of these models have been expressed as relatively simple empirical relations covering the full range of λ_+_/Λ and λ_De_/Λ (or *n* Λ and *J/n*^2^). When a magnetic field is present models of experiment are available for all λ_+_/Λ and ω*_B_/ν*_in_ (*n*Λ and *B/n*), but only for λ_De_/Λ<< 1 (or small *J/n*^2^). With a magnetic field the experimental tests show a high sensitivity to boundary conditions and a propensity of the plasma to become unstable. Empirical relations connecting the magnetic field and other diffusion parameters have not been developed.

In many practical applications it is necessary to include nonlinear processes such as electron-excited state or electron-ion collisions in a complete plasma model. In such cases one expects degradation of the accuracy of the empirical formulas used to represent the diffusion contribution. The error in such an approximation is usually small when calculating the average rate of charge particle loss because the contribution of diffusion is decreasing as the other processes become more important. However, large errors can occur when these relations are used in the calculation of the flux of charged particles to the boundaries and when competing loss processes, such as electron-ion recombination, significantly alter the spatial distribution of the charged particles.
